# Distinct Community Assembly Processes of Abundant and Rare Soil Bacteria in Coastal Wetlands along an Inundation Gradient

**DOI:** 10.1128/mSystems.01150-20

**Published:** 2020-12-22

**Authors:** Gui-Feng Gao, Dan Peng, Binu M. Tripathi, Yihui Zhang, Haiyan Chu

**Affiliations:** a State Key Laboratory of Soil and Sustainable Agriculture, Institute of Soil Science, Chinese Academy of Sciences, Nanjing, China; b Key Laboratory of the Ministry of Education for Coastal and Wetland Ecosystems, College of the Environment and Ecology, Xiamen University, Xiamen, China; c Korea Polar Research Institute, Incheon, Republic of Korea; d University of Chinese Academy of Sciences, Beijing, China; University of Delhi

**Keywords:** rare bacteria, abundant bacteria, assembly processes, plant biomass, coastal wetlands

## Abstract

Microbial communities commonly consist of a large number of rare taxa (RT) and few abundant taxa (AT), and it is important to identify the differences of the community assembly processes between RT and AT in response to environmental changes. However, the community assembly processes governing AT and RT in coastal wetland soils along an inundation gradient remain elusive. Here, an *in situ* mesocosm, with continuous inundation gradients and native mangrove Kandelia obovata or exotic cordgrass Spartina alterniflora, was established to determine the patterns and driving factors of community turnover and assembly processes of AT and RT. We found that RT exhibited a remarkably lower turnover rate than AT, and the niche breadth of RT was significantly narrower than that of AT. In comparison with AT, RT presented stronger phylogenetic signals for ecological preferences across environmental gradients. Null model analyses revealed that RT were more phylogenetically clustered and primarily governed by homogeneous selection, while AT were more overdispersed and dominated by dispersal limitation. Soil water content was the most decisive factor for community turnover and assembly processes of both AT and RT. Structural equation modeling analysis showed that RT were strongly associated with K. obovata biomass rather than S. alterniflora biomass, suggesting a strong relationship between RT and the growth of mangrove K. obovata. Overall, our study revealed distinct assembly processes of soil AT and RT communities in coastal wetlands, which is crucial for mechanistic understanding of the establishment and maintenance of soil microbial diversity in coastal wetlands under conditions of global environmental changes.

**IMPORTANCE** Coastal wetlands are one of the important ecosystems that play a crucial role in the regulation of climate change. Rare taxa (RT) exist in one habitat along with abundant taxa (AT). In this study, we found that RT exhibited narrower niche breadth and stronger phylogenetic signals than AT. Null model analyses showed that RT were more phylogenetically clustered and primarily governed by homogeneous selection, while AT were more overdispersed and dominated by dispersal limitation. Revealing the differences in the community assembly processes between AT and RT in coastal wetlands is critical to understand the establishment and maintenance of soil microbial diversity in coastal wetlands with regard to environmental changes.

## Abstract (precis)

Coastal wetlands are one of the important ecosystems that play a crucial role in the regulation of climate change. Rare taxa (RT) exist in one habitat along with abundant taxa (AT).

## INTRODUCTION

One of the central objectives of microbial ecology is to determine the relative contributions of deterministic processes (e.g., variable selection) and stochastic processes (e.g., dispersal limitation) in microbial community assembly (1, 2). Understanding the fundamental mechanisms of the establishment and maintenance of microbial diversity is crucial for unraveling the relationships between microbial communities and ecosystem functions and for deciphering the responses and feedbacks of microbial communities to environmental changes (2–4).

Microbial communities typically exhibit a skewed distribution of species abundance, with a small number of abundant taxa (AT) and a large number of rare taxa (RT) coexisting in one habitat (5, 6). Previous studies have demonstrated the crucial role of RT in governing the functions of the microbiome, such as participating in biogeochemical cycles and facilitating plant growth (7–10). Determining the differences in the community assembly processes between RT and AT is critical to understand the responses of a rare biosphere to environmental changes. Recently, such differences in different ecosystems, including agricultural fields (11–13), subtropical bays (14, 15), and Tibetan Plateau grassland (16), have been widely studied. These studies showed that AT and RT generally exhibit distinct distribution patterns and assembly processes. For instance, it was found that the abundance of AT, instead of RT, is mainly limited by the dispersion process in agricultural fields across eastern China (11, 13) and in inland freshwater ecosystems in China (17). On the contrary, other studies have revealed that the abundance of RT is mostly limited by dispersion in subtropical bays (14) and in cascade reservoirs of the Jinsha River in China (18). Environmental factors strongly influence the balance between stochastic and deterministic processes of community assembly (1, 19, 20). For example, Tripathi et al. revealed that extreme soil pH could result in deterministic assembly of soil bacterial communities, while neutralized soil pH could lead to stochasticity during both short- and long-term succession stages (20). In addition, the changes of organic matters in soil also influence the stochastic and deterministic processes, which can shape soil bacterial communities in agroecosystems across subtropical China (19). These indicate that the relative dominance of assembly processes that structure AT and RT is varied in different ecosystems, which could be mediated by different environmental factors across both spatial and temporal scales. However, to date, little is known about the community assembly processes of AT and RT and their driving factors in coastal wetlands with environmental gradients.

The responses of microorganisms toward environmental changes tend to be phylogenetically conserved (21). For example, the influence of pH in the global bacterial biogeographic distribution exhibits strong phylogenetic conservation (21, 22). Therefore, revealing the phylogenetic conservatism of microbial response traits can facilitate the prediction of their evolutionary adaptation in response to environmental changes. However, the phylogenetic distribution of microbial communities, particularly AT and RT, in coastal wetlands has not been reported.

Coastal wetlands, located in the intertidal areas across a broad range of inundation gradients, are important ecosystems with valuable functions (23–25). They experience periodic tides and are characterized by aerobic-anaerobic fluctuations and high salinity (26). In coastal wetlands, inundation frequency is one of the most important environmental factors that influence soil microbial community (27, 28) and plant growth (29, 30). Due to global environmental changes, such as sea level rise and seawater intrusion, coastal wetlands are facing prolonged flooding, which intensively impacts their ecological functions. Therefore, understanding the microbial community assembly processes along inundation gradients is important. Recent studies have reported that coastal wetlands harbor a unique soil microbial community that is fundamentally different from that in other ecosystems (31). Thus, we hypothesize that the assembly processes of AT and RT communities in coastal wetlands are different from those in other biomes. Spartina alterniflora, originally from North America, was first introduced to China in 1979 and has rapidly spread along the eastern coastlines of China over the past 4 decades (32, 33). Currently, S. alterniflora and native mangrove plant species, such as Kandelia obovata, are commonly found to coexist in most coastal wetlands in southeastern China. K. obovata is regarded as a nutrient-limited species in subtropical coastal wetlands (34), while S. alterniflora has a strong environmental adaptability and a high degree of phenotypic plasticity (35–37). In China, the invasion of S. alterniflora into mangroves can change soil microbial community, ultimately resulting in large differences in the associated ecological functions and responses toward environmental changes (38, 39). Nevertheless, little is known about the impact of S. alterniflora invasion on microbial community assembly in mangroves, which compromises the understanding and prediction of the effects of S. alterniflora invasion on mangrove soil microbial communities.

In this study, an *in situ* mesocosm with continuous inundation gradients and planting of K. obovata and S. alterniflora monocultures was applied to evaluate the assembly processes of AT and RT. In addition, we examined the niche breadth and phylogenetic signals for the ecological preferences across environmental gradients for both AT and RT. Considering the low competition capacity, slow growth (9, 40), and the important role of RT in soil nutrient cycling and plant growth (8, 10, 41), we aimed to investigate (i) if there is any difference in the relative influence of assembly processes that govern the composition of AT and RT in a coastal wetland ecosystem along an inundation gradient, and (ii) if the relationship between RT and plant biomass varies according to plant species. Overall, our findings aid in understanding the mechanism of the establishment and maintenance of microbial diversity in coastal wetland ecosystems and in predicting the responses of soil microbial communities to global environmental changes.

## RESULTS

### General responses of AT and RT to environmental changes.

In total, 17,634 amplicon sequence variants (ASVs) from 2,918,880 valid sequences were obtained, among which, 863 and 16,771 ASVs were classified into AT and RT, accounting for 4.89% and 95.11% of the total ASVs, respectively (see Table S1A in the supplemental material). The rarefaction curves for all of the soil samples showed a decreasing trend in the observed ASVs against the sequence numbers and eventually reached the plateau phase (see Fig. S1). Spearman’s rank correlation showed that both AT and RT exhibited a strong abundance-occupancy relationship (see Fig. S2A). We also observed strong correlations between taxa’s niche widths and their relative abundances for both AT and RT (Fig. S2B). RT contributed to 51.60% and 50.73% of the shifts of bacterial beta diversity in K. obovata and S. alterniflora soils, respectively, and the contribution of RT remained relatively stable under different contents of soil water (Fig. S2C and D). The most abundant phylum in both AT and RT was *Proteobacteria*, which tended to decrease along an increased elevation. In addition, the relative abundances of several phyla, such as *Bacteroidetes* and *Planctomycetes*, were found to be higher in RT than in AT (see Fig. S3).

**FIG 1:**
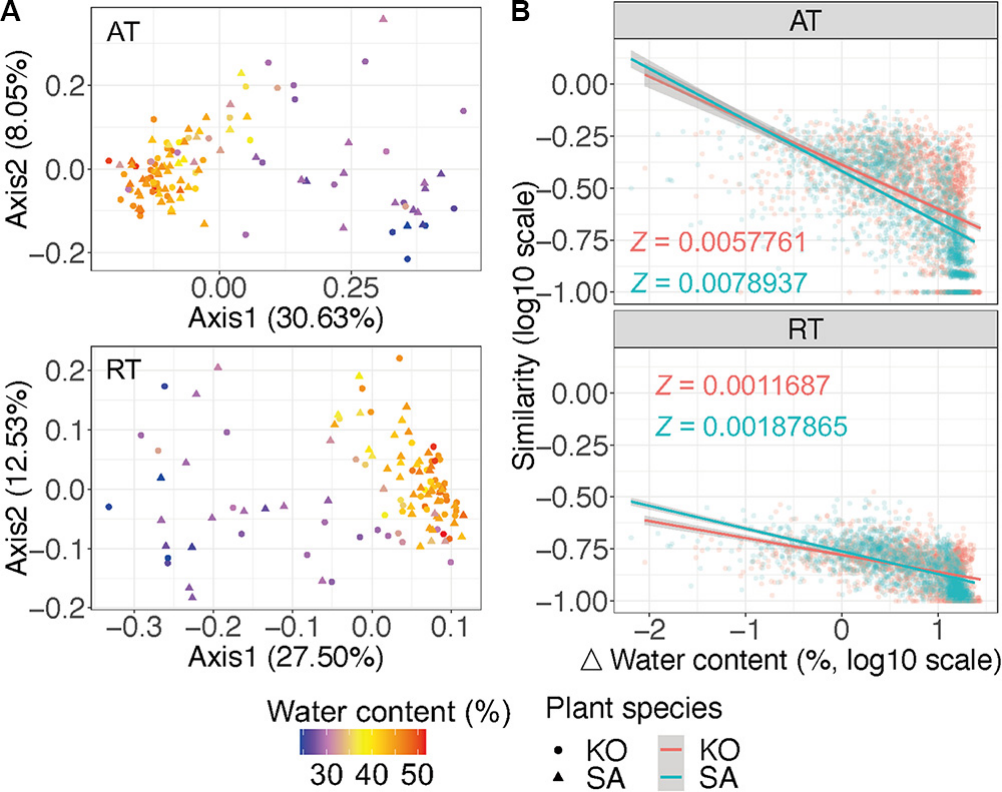
(A) Principal-coordinate analyses (PCoAs) of the bacterial communities along soil water content gradients for AT and RT of K. obovata and S. alterniflora soils. (B) Distance-decay relationship of AT and RT of K. obovata and S. alterniflora soils between Sørensen similarity and distance in soil water content. AT, abundant taxa; RT, rare taxa; KO, *Kandelia obovata*; SA, Spartina alterniflora.

10.1128/mSystems.01150-20.1 TABLE S1 (A) General descriptions of the whole bacterial community (All), abundant taxa (AT), rare taxa (RT), and moderate taxa (MT) datasets. (B) Results of pairwise permutational multivariate analysis of variance (PERMANOVA) showing the effects of elevation and plant on bacterial communities. (C) Mantel tests examining the significance of the relationships between soil physicochemical properties and bacterial communities. (D) Two-way ANOVAs showing the effects of elevation, plant species, and their interactions on the alpha diversity. (E) Spearman’s correlations between soil physicochemical properties and the alpha diversity. (F) Relationship between changes in soil water content and the Sørensen similarity. (G) *P* values of standardized major axis regression analysis evaluating the significant difference of the slopes of distance-decay relationship. (H) Mantel tests examining the significance of the relationships between soil physicochemical properties and betaNTI. For abbreviations, see legend of Fig. 1. Download Table S1, XLSX file, 0.1 MB. Copyright © 2020 Gao et al. 2020 Gao et al. This content is distributed under the terms of the Creative Commons Attribution 4.0 International license.

10.1128/mSystems.01150-20.2 FIG S1 Rarefaction curves showing the observed amplicon sequence variants (ASVs) with the changes of sequence numbers for all of the soil samples. For abbreviations, see legend of Fig. 1. Download FIG S1, PDF file, 0.5 MB. Copyright © 2020 Gao et al. 2020 Gao et al. This content is distributed under the terms of the Creative Commons Attribution 4.0 International license.

10.1128/mSystems.01150-20.3 FIG S2 Abundance-occupancy relationship of bacterial taxa. (a) Spearman’s rank correlation between the log-transformed mean relative abundance of bacteria and number of sites occupied. *N* is the number of ASVs. (B) Niche breadth of bacterial taxa. Relative abundance was log_10_ transformed. (C) Comparison of the fraction of beta diversity attributed to RT community between K. obovata and S. alterniflora soils. (D) Changes in the fraction of beta diversity attributed to RT community along soil water content gradients. For abbreviations, see legend of Fig. 1. Download FIG S2, TIF file, 2.6 MB. Copyright © 2020 Gao et al. 2020 Gao et al. This content is distributed under the terms of the Creative Commons Attribution 4.0 International license.

10.1128/mSystems.01150-20.4 FIG S3 Relative abundance of top 10 phyla for AT and RT communities under different elevations. For abbreviations, see legend of Fig. 1. Download FIG S3, PDF file, 0.1 MB. Copyright © 2020 Gao et al. 2020 Gao et al. This content is distributed under the terms of the Creative Commons Attribution 4.0 International license.

We further explored the main environmental factors that influenced the soil bacterial communities. Pairwise permutational multivariate analysis of variance (PERMANOVA) revealed that elevation significantly affected both AT (*F *=* *6.76, *P* < 0.001) and RT (*F *=* *6.48, *P* < 0.001), while plant species only influenced RT (*F *=* *2.18, *P* = 0.03) (Table S1B). This result was also supported by the variation partitioning analysis (VPA), which suggested that AT and RT were influenced by soil physicochemical properties rather than plant species (see Fig. S4). Meanwhile, Mantel tests also demonstrated that both AT and RT were strongly associated with multiple soil physicochemical properties, including soil water content, salinity, total carbon (TC), inundation, and others (Table S1C). Among them, soil water content was the most decisive factor in regulating the bacterial community structures of AT (*r *=* *0.54, *P* < 0.001) and RT (*r *=* *0.54, *P* < 0.001).

10.1128/mSystems.01150-20.5 FIG S4 Variation partition analysis (VPA) of the effects of plant and soil physicochemical properties on AT and RT communities. For abbreviations, see legend of Fig. 1. Download FIG S4, PDF file, 0.1 MB. Copyright © 2020 Gao et al. 2020 Gao et al. This content is distributed under the terms of the Creative Commons Attribution 4.0 International license.

Indices of observed ASVs and Faith’s phylogenetic diversity (Faith’s PD) were used to present the taxonomic and phylogenetic diversity, respectively. Two-way analysis of variance (ANOVA) revealed that the observed ASVs of AT were mainly influenced by elevation (*F *=* *5.19, *P* < 0.001), while the observed ASVs of RT were mainly affected by plant species (*F *=* *17.37, *P* < 0.001) (Table S1D). In addition, Faith’s PDs of both AT and RT were influenced by either elevation or plant species, the latter of which had a more profound influence (Table S1D). The observed ASVs and Faith’s PD of RT were significantly (*P* < 0.001) higher than those of AT, in both K. obovata and S. alterniflora soils (see Fig. S5A). In addition, the alpha diversity indices in S. alterniflora soil, except for the observed ASVs of AT (*P* = 0.60), were all significantly (*P* < 0.05) higher than those in K. obovata soil (Fig. S5A). Bacterial alpha diversity of AT and RT was strongly correlated with soil physicochemical properties (Table S1E). For AT, increasing soil water content significantly decreased the observed ASVs in both K. obovata (ρ = −0.27, *P* = 0.04) and S. alterniflora (ρ = −0.25, *P* = 0.04) soils (Fig. S5B). Similarly, the Faith’s PDs of K. obovata (*rho* = −0.69, *P* < 0.001) and S. alterniflora (ρ = −0.41, *P* = 0.001) soils were significantly and negatively correlated with soil water content (Fig. S5B). However, for RT, only the Faith’s PD of S. alterniflora soil was significantly correlated with soil water content (ρ* *=* *0.30, *P* = 0.02) (Fig. S5B).

10.1128/mSystems.01150-20.6 FIG S5 (A) Boxplots showing the differences of the alpha diversity of AT and RT communities between K. obovata and S. alterniflora soils. Significant differences were examined by Wilcoxon rank sum test. (B) Relationship between soil water content and the alpha diversity of AT and RT communities. Significance of the relationship was examined by Spearman’s ρ correlation. Significant and insignificant relationships are showed as solid and dashed lines, respectively. The significant difference between two slopes was analyzed and compared by standardized major axis (SMA) regression analysis. For abbreviations, see legend of Fig. 1. Download FIG S5, PDF file, 0.2 MB. Copyright © 2020 Gao et al. 2020 Gao et al. This content is distributed under the terms of the Creative Commons Attribution 4.0 International license.

### Distance-decay relationship and community turnover.

Principal-coordinate analysis (PCoA) revealed that AT and RT were noticeably divided along the gradients of soil water content on axes 1 and 2, with 30.63% and 27.50% interpretations on axis 1, respectively (Fig. 1A). Significant (*P* < 0.001) distance-decay relationships were also observed in AT and RT of both K. obovata and S. alterniflora soils (Fig. 1B). We further calculated the rate of community turnover (*Z* value) (Table S1F). Standardized major axis (SMA) regression analyses revealed that the slope of distance-decay relationship in RT was significantly (*P* < 0.001) lower than that in AT, suggesting that RT had a lower rate of community turnover (Table S1F and G). In addition, the *Z* values for both AT and RT in S. alterniflora soil were significantly (*P* < 0.001) higher than those in K. obovata soil, indicating that bacterial community in S. alterniflora soil was more responsive to the changes of soil water content (Fig. 1B; Table S1F and G).

**FIG 2:**
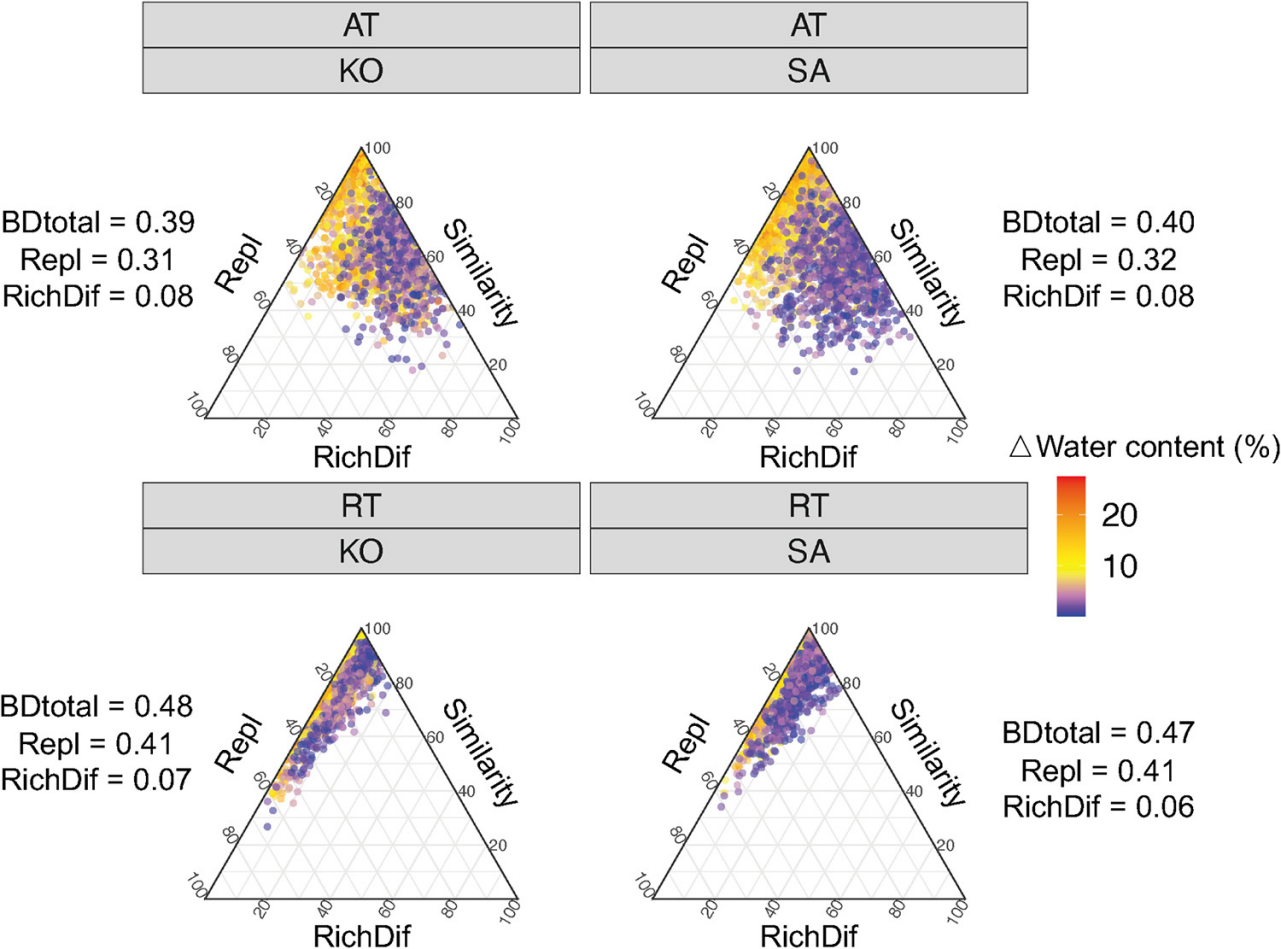
Ternary plots of beta diversity components for AT and RT of K. obovata and S. alterniflora soils. Each point represents a value and is colored according to the differences in soil water content between two samples. The position of each point is determined by a triplet of values from similarity, Repl and RichDif matrices. BDtotal, total beta diversity; Repl, total replacement diversity; RichDif, total richness difference diversity. For abbreviations, see legend of Fig. 1.

The bacterial beta diversity was further partitioned into total replacement diversity (Repl) and total richness difference diversity (RichDif). We found that the dissimilarity of bacterial community compositions of AT and RT for both S. alterniflora and K. obovata soils were dominated by species replacement processes (Fig. 2). The total beta diversity (BDtotal) of RT (K. obovata, 0.48; S. alterniflora, 0.47) was higher than that of AT (K. obovata, 0.39; S. alterniflora, 0.40). RichDif accounted for a larger proportion of BDtotal (up to 71.93% in AT), especially when the difference of soil water content was small, suggesting that intensive changes of soil water content could induce species replacement (Fig. 2).

### Environmental adaption of AT and RT.

**FIG 3:**
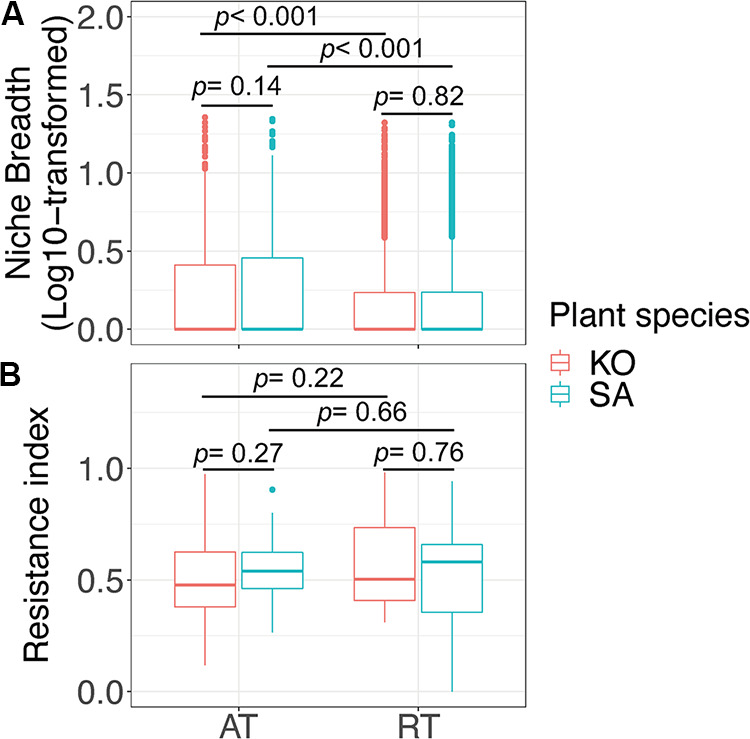
Niche breadths (log_10_ transformed) (A) and resistance indices (B) of AT and RT of K. obovata and S. alterniflora soils. Significant differences were examined by Wilcoxon rank sum test. For abbreviations, see legend of Fig. 1.

**FIG 4:**
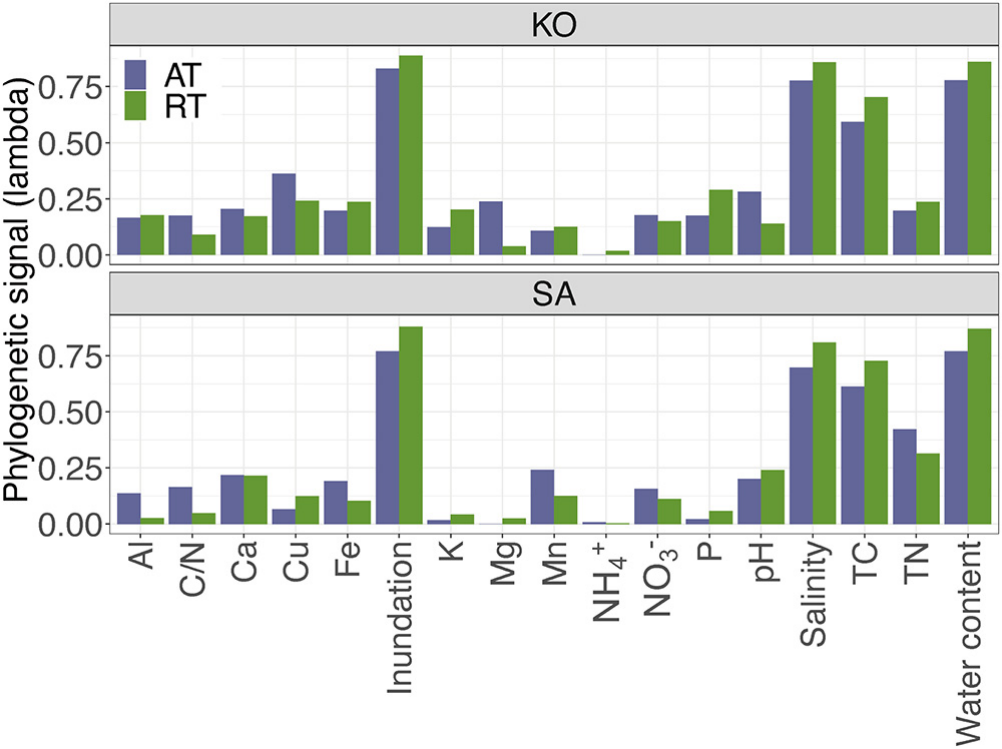
Phylogenetic signals (lambda) showing the trait conservatism of AT and RT of K. obovata and S. alterniflora soils for each soil physicochemical property. For abbreviations, see legend of Fig. 1.

We characterized the environmental adaptations of bacterial communities by using three indices: Levin’s niche breadth index, Orwin-Wardle resistance index, and Pagel’s lambda (λ) phylogenetic signal (Fig. 3 and 4). For both K. obovata and S. alterniflora soils, the niche breadth index of AT was significantly (*P* < 0.001) higher than that of RT (Fig. 3A). However, no significant difference was found in the niche breadth indices for both AT (*P* = 0.14) and RT (*P* = 0.82) between K. obovata and S. alterniflora soils. In addition, we also found that the resistance index of RT was slightly higher than that of AT, though at an insignificant level (*P* > 0.22) (Fig. 3B).

The phylogenetic signal was next examined, and we found that both AT and RT exhibited strong phylogenetic signals, indicating a significant influence of phylogenetic history on the ecological traits of the soil bacterial community (Fig. 4). RT exhibited stronger phylogenetic signals for most of the environmental variables than AT, including soil water content, TC, salinity, inundation, and others, which indicated that closely related bacterial taxa of RT exhibited more similar ecological preferences in response to environmental changes.

### Bacterial community assembly processes.

**FIG 5:**
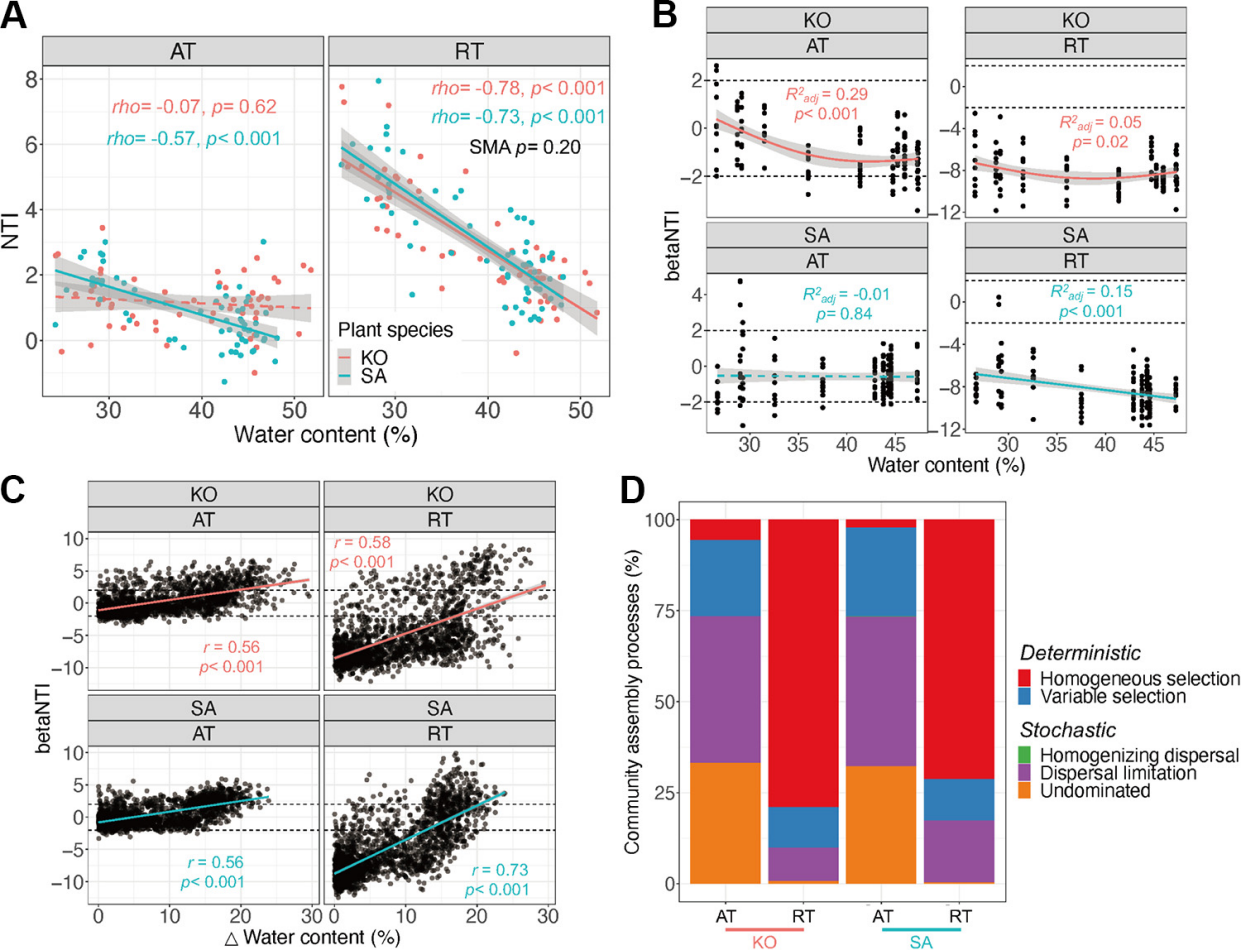
(A) Relationship between soil water content and nearest taxon index (NTI). Significance of the relationship was examined by Spearman’s ρ correlation. (B) Beta nearest taxon index (betaNTI) value under different soil water content regimes. The horizontal dashed lines indicate the betaNTI values of +2 and −2. Data were fitted using generalized additive model. (C) Relationship between changes in soil water content and betaNTI. Data were fitted using linear regression and examined by the Mantel test. (D) Relative contribution of each ecological process to community assembly. Significant and insignificant relationships are shown as solid and dashed lines, respectively. For abbreviations, see legend of Fig. 1.

The phylogenetic mantel correlogram revealed significant (*P* < 0.05) phylogenetic signals across short phylogenetic distances, indicating that the ecological traits in regulating bacterial community assembly processes were phylogenetically conserved (see Fig. S6A and B). Mantel tests demonstrated that the assembly processes were significantly (*P* < 0.01) associated with certain environmental variables, including soil water content, salinity, TC, inundation, and others (Table S1H). Among these variables, soil water content was the most decisive factor in regulating bacterial community assembly for both AT (K. obovata: Mantel *r *=* *0.56, *P* < 0.001; S. alterniflora: Mantel *r *=* *0.56, *P* < 0.001) and RT (K. obovata: Mantel *r *=* *0.58, *P* < 0.001; S. alterniflora: Mantel *r *=* *0.73, *P* < 0.001). Moreover, the nearest taxon index (NTI) value of RT was significantly (*P* < 0.001) higher than that of AT, suggesting a more phylogenetic clustering in RT (Fig. S6C). We also found that the NTI value, except for that of AT in K. obovata soil (ρ = −0.07, *P* = 0.62), was significantly and negatively correlated with soil water content, indicating that the increased soil water content could reduce the level of phylogenetic clustering (Fig. 5A).

10.1128/mSystems.01150-20.7 FIG S6 Phylogenetic Mantel correlogram showing significant phylogenetic signals across short phylogenetic distances for AT (A) and RT (B), indicating that bacterial ecological preferences are strongly phylogenetically conserved across short phylogenetic distances. Closed and open symbols indicate significant (*P < *0.05) and insignificant (*P > *0.05) correlations, respectively. (C) Boxplot showing the differences of nearest taxon indices (NTIs) of AT and RT communities between K. obovata and S. alterniflora soils. Significant differences were examined by Wilcoxon rank sum test. For abbreviations, see legend of Fig. 1. Download FIG S6, PDF file, 0.1 MB. Copyright © 2020 Gao et al. 2020 Gao et al. This content is distributed under the terms of the Creative Commons Attribution 4.0 International license.

Furthermore, most of the beta nearest taxon index (betaNTI) values of AT were between −2 and 2, indicating the dominant role of stochastic processes (dispersal limitation and undominated processes) in AT community assembly (Fig. 5B). The betaNTI values of AT in K. obovata soil, rather than in S. alterniflora soil, were significantly (adjusted *R*^2^ [*R*_adj_^2^] = 0.29, *P* < 0.001) and negatively correlated with soil water content (Fig. 5B). On the contrary, most of the betaNTI values of RT were less than −2, suggesting that the bacterial community assembly was mainly governed by deterministic processes (homogeneous selection) (Fig. 5B). The betaNTI values of RT in both S. alterniflora (*R*_adj_^2^ = 0.15, *P* < 0.001) and K. obovata (*R*_adj_^2^ = 0.05, *P* = 0.02) soils were significantly correlated with soil water content (Fig. 5B). We observed that the betaNTI was significantly (*P* < 0.001) correlated with the changes of soil water content, facilitating the transition of bacterial community assembly from homogeneous selection to stochastic processes and further to variable selection (Fig. 5C). The quantitative estimates of the relative contribution of assembly processes showed that stochastic processes were dominant in AT, while deterministic processes were dominant in RT (Fig. 5D).

### Correlations between soil bacterial community and plant biomass.

**FIG 6:**
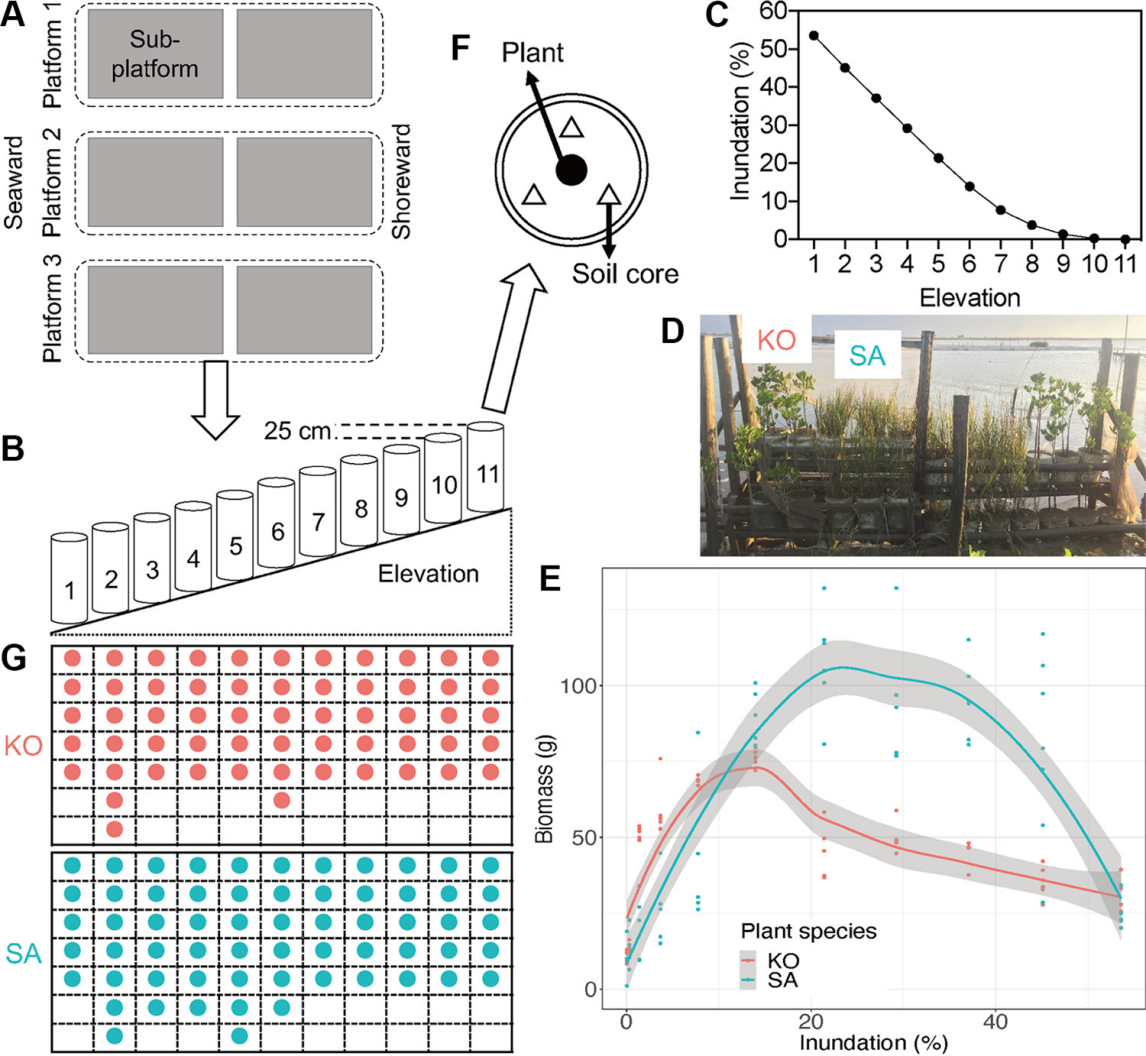
Experimental design. (A) Location of each platform. (B) Elevation establishment of the *in situ* mesocosm. (C) Changes in inundation frequency along different elevation gradients. (D) Growth of K. obovata and S. alterniflora in a subplatform in September 2018. (E) Changes of biomass of K. obovata and S. alterniflora under different inundation treatments. (F) Sample strategy of soils from each bucket. (G) Biological replicates of each elevation for K. obovata and S. alterniflora soil. For abbreviations, see legend of Fig. 1.

**FIG 7:**
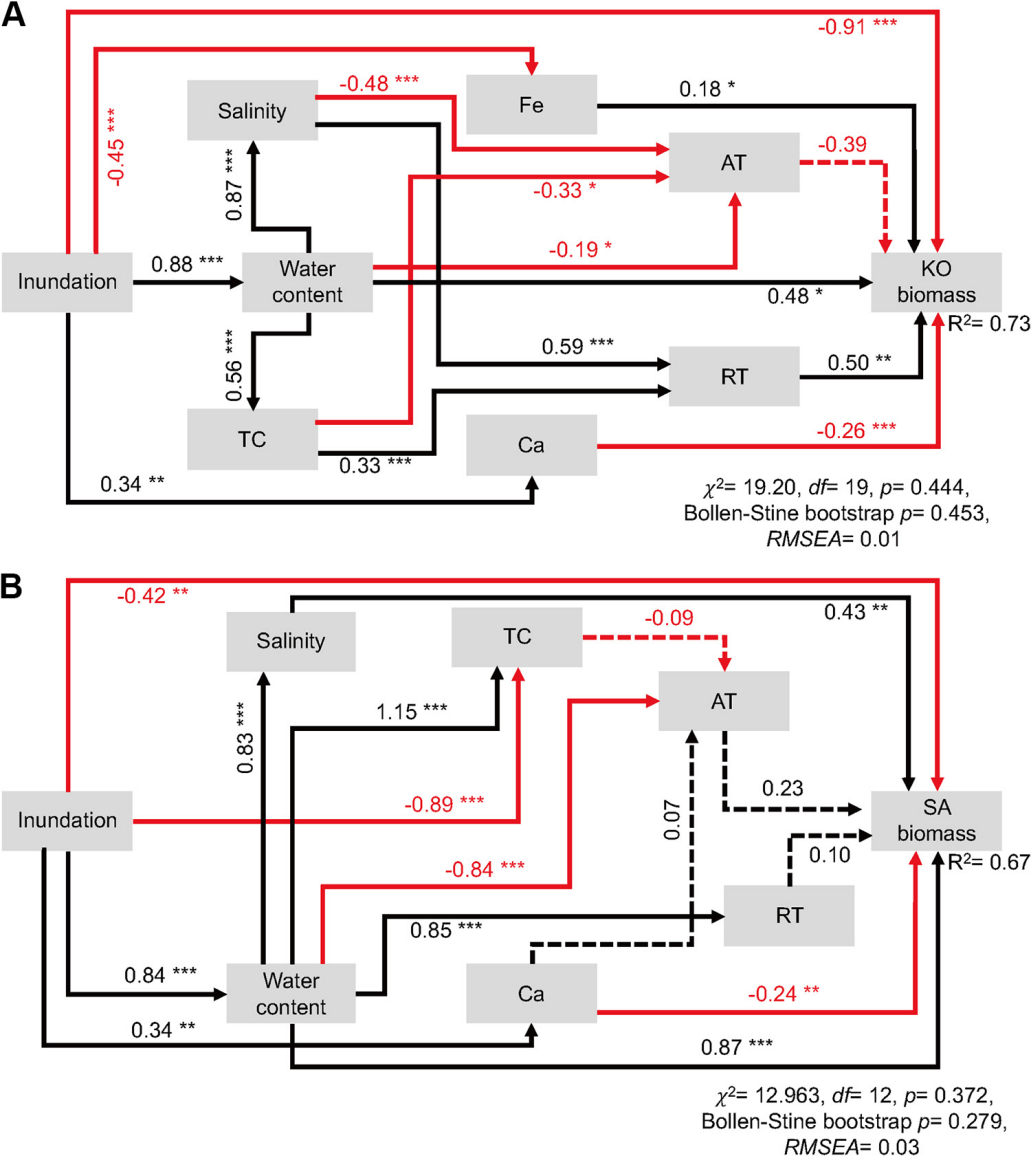
Structural equation model (SEM) revealing the direct and indirect effects of soil physicochemical properties, AT, and RT on K. obovata (A) and S. alterniflora (B) biomass. Numbers on arrows are the path coefficients and are indicative of the standardized effect size of the relationship. Arrows in black and red indicate positive and negative effects, respectively. *R*^2^ means the proportion of variance explained. Solid and dashed lines indicate significant and insignificant correlations, respectively. *, *P* < 0.5; **, *P* < 0.01; ***, *P* < 0.001. For abbreviations, see legend of Fig. 1.

In order to investigate the direct and indirect effects of increasing inundation on plant biomass with simultaneous consideration of multiple factors, we used a structural equation model (SEM) to assess the potential correlations among environmental factors, bacterial community structure, and plant biomass. We found that both K. obovata and S. alterniflora displayed a hump-shaped pattern of biomass across inundation gradients (Fig. 6E). K. obovata performed the best at slightly higher elevations (with an inundation frequency between 7.73% and 13.90%), while S. alterniflora performed the best at slightly lower elevations (with an inundation frequency between 21.40% and 29.20%) (Fig. 6E). First, we identified the most important factors that influenced the biomass of K. obovata and S. alterniflora as well as the bacterial communities of AT and RT (see Fig. S7 and S8), which explained 73% and 67% of the total variation of K. obovata and S. alterniflora biomass, respectively (Fig. 7). The SEM revealed that K. obovata biomass was significantly and positively correlated with RT (λ = 0.50, *P* < 0.01), water content (λ = 0.48, *P* < 0.05), and Fe content (λ = 0.18, *P* < 0.05) while significantly and negatively correlated with inundation (λ = −0.91, *P* < 0.001) and Ca content (λ = −0.26, *P* < 0.001) (Fig. 7A). However, S. alterniflora biomass was significantly and positively correlated with salinity (λ = 0.43, *P* < 0.01) and water content (λ = 0.87, *P* < 0.001) and significantly and negatively correlated with inundation (λ = −0.42, *P* < 0.01) and Ca content (λ = −0.24, *P* < 0.01) but insignificantly (*P* > 0.05) correlated with either AT or RT (Fig. 7B). Overall, based on the total effects that were standardized from SEM, it was suggested that K. obovata biomass was primary influenced by soil water content, RT, and salinity, while S. alterniflora biomass was mainly driven by soil water content, inundation, and salinity (see Fig. S9).

10.1128/mSystems.01150-20.8 FIG S7 Classification random forest analyses were conducted to identify the most important predictors in influencing K. obovata seeding biomass (A), community structure of AT (B), and community structure of RT (C). The predictors marked in red and black indicate significant (*P*< 0.05) and insignificant (*P* > 0.05) after 1,000 permutations, respectively. For abbreviations, see legend of Fig. 1. Download FIG S7, PDF file, 0.1 MB. Copyright © 2020 Gao et al. 2020 Gao et al. This content is distributed under the terms of the Creative Commons Attribution 4.0 International license.

10.1128/mSystems.01150-20.9 FIG S8 Classification random forest analyses were conducted to identify the most important predictors in influencing S. alterniflora seeding biomass (A), community structure of AT (B), and community structure of RT (C). The predictors marked in red and black indicate significant (*P* < 0.05) and insignificant (*P* > 0.05) after 1,000 permutations, respectively. For abbreviations, see legend of Fig. 1. Download FIG S8, PDF file, 0.1 MB. Copyright © 2020 Gao et al. 2020 Gao et al. This content is distributed under the terms of the Creative Commons Attribution 4.0 International license.

10.1128/mSystems.01150-20.10 FIG S9 Standardized total effects of each predictor attribute on K. obovata and S. alterniflora seeding biomass. For abbreviations, see legend of Fig. 1. Download FIG S9, PDF file, 0.1 MB. Copyright © 2020 Gao et al. 2020 Gao et al. This content is distributed under the terms of the Creative Commons Attribution 4.0 International license.

## DISCUSSION

### Distinct assembly processes of AT and RT.

Quantifying the relative contributions of deterministic and stochastic processes on community assembly is one of the central objectives of microbial ecological studies (1, 5, 42). In this study, we found that deterministic processes primarily governed RT, while stochastic processes dominated AT in coastal wetlands (Fig. 5). Our findings were consistent with previous studies which have reported the dominance of deterministic processes in RT, rather than in AT, in agricultural fields across eastern China (11, 13) and the northwestern Pacific Ocean (43). It is possible that the habitat occupancy of AT with a wide niche breadth is more likely to be limited by dispersion, whereas the distribution of RT with a narrow niche breadth might be delimited by environmental filtering. On the contrary, several studies have revealed that the assembly processes of RT were primarily limited by neutral processes, in comparison with those of AT, in subtropical bays (14) and cascade reservoirs of the Jinsha River in China (18). Such discrepancies of microbial community assembly could be partially attributed to spatial scale dependency and habitat diversity (44). Moreover, these inconsistent findings might also be explained by the variations in metabolic activity, body size, and dispersal potential among different microbial populations in soil (45, 46).

Understanding the ecological niches and evolutionary characteristics of microbial ecological traits is important for determining the mechanism of community assembly (11, 47). Here, we further investigated the differences of environmental adaptive capabilities between AT and RT in coastal wetlands. First, we found that the niche breadth of RT was narrower than that of AT (Fig. 3). It is generally recognized that AT can efficiently utilize a wider range of resources than RT, thus becoming more abundant in the same environment (5, 11). Thus, the AT can be easily dispersed, as there are more individuals in the environment (17). On the contrary, the soil bacteria in RT were unevenly distributed in limited locations, with relatively low abundances. The restricted niche breadth of RT may be attributed to the low competitive capacity and growth rate (9, 40). Overall, the narrower niche breadth of RT than that of AT can induce increased competition among different microorganisms for similar resources, where environmental filtering plays a dominant role in microbial community assembly processes. Nevertheless, experimental verifications of microbial taxa in response to environmental changes are essential for environmental management, since the niche breadth analyses based on statistics might remain discrepant with the real situation.

Second, environmental adaptation could be partially reflected by phylogenetic conservation of the traits for microbial ecological preferences. Phylogenetic conservation of traits could provide projections on the evolutionary adaptation of microbial communities subject to ongoing environmental changes (21). In the present study, we found that RT exhibited stronger phylogenetic signals for most of the ecological preferences than AT, particularly for the main factors influencing bacterial community, such as soil water content, salinity, inundation, and TC (Fig. 4). In the presence of significant phylogenetic signals, environmental filtering could result in phylogenetic clustering within the community (48, 49). Accordantly, we also found that AT had a significantly lower NTI than RT, indicating that the soil bacteria in RT were more phylogenetically clustered (Fig. 5A; see also Fig. S6C in the supplemental material). In fact, previous studies have demonstrated that deterministic processes are positively associated with phylogenetic clustering in bacterial communities (20, 50).

Microbial community assembly is mediated by the balance between stochastic and deterministic processes (5, 42). We found a significantly lower rate of community turnover in RT than in AT (Fig. 1), which was consistent with prior studies based on RT in the Yangtze River basin (51) as well as in lakes and reservoirs across China (14, 17, 18). On one hand, RT can become dominant under favorable environmental conditions (7), evolve to adapt to environmental changes, and increase the community resistance to environmental disturbances (Fig. 3). On the other hand, the assembly processes of RT were dominated by homogeneous selection in comparison with the dominancy of variable selection and dispersal limitation in AT assembly processes, which enabled RT with greater convergence and weaker distance-decay relationships (4, 19).

Additionally, we also observed that the community turnover rates for both AT and RT were significantly higher in S. alterniflora soil than in K. obovata soil (Fig. 1). This might be due to the higher variable selection and dispersal limitation in the community assembly processes of S. alterniflora soil, which increase the divergence in the microbial community composition. The above-mentioned results also suggested that the bacterial community in S. alterniflora soil is more responsive to environmental changes. Currently, S. alterniflora is rapidly spreading and occupying most of the coastal wetlands in China, which induces significant changes in the underground microbial communities and their ecological functions (33). The present study facilitates an in-depth mechanistic understanding of the responses of soil bacterial community to environmental changes in coastal wetlands.

### RT is important for mangrove plant performance.

The rare biosphere plays an important role in mediating soil nitrogen cycling (8, 10, 41), pollutant degradation (52), and plant growth (10, 53, 54). We have observed a strong association between RT and K. obovata biomass (Fig. 7), which is consistent with previous studies suggesting the importance of soil RT in regulating the aboveground biomass in alpine grassland in Qinghai-Tibetan Plateau (10). Such an association might be due to the crucial functions of RT in soil carbon cycling (55, 56), nitrogen fixation (8), and control of plant pathogens (57). Past studies have reported that RT can be more active than AT (9). Furthermore, previous studies have reported that under fluctuating conditions, such as in coastal areas experiencing periodic tides, RT could become hyperactive as a result of continuous regrowth (58). Moreover, the huge functional gene pool in RT could contribute to the outstanding metabolic potential, especially unique metabolic pathways, for the microbial communities under appropriate conditions (59). Additionally, the functionality of RT could also be enhanced by AT through microbial interactions (60). However, the current findings were mainly derived from the SEM analyses, for which the specific microbial taxa at play were unknown. Further isolation and functional studies on these key microbial taxa by multi-omics will contribute to a deeper understanding of mangrove growth in response to environmental changes.

In contrast, a strong relationship between RT and S. alterniflora biomass was not observed (Fig. 7), which might be due to the different plant adaptive strategies between native species K. obovata and invasive species S. alterniflora. S. alterniflora is an exotic perennial C_4_ cordgrass and has a strong environmental adaptability (37). Previous studies have demonstrated that the invasive ability of S. alterniflora in China is mostly governed by provenance-by-environment interactions (35, 61, 62). Thus, the rapid growth and spread of S. alterniflora are possibly attributed to its broad capacity of preadaptation and high degree of phenotypic plasticity (35, 36). Moreover, compared with K. obovata, S. alterniflora is more tolerant to prolonged inundation (Fig. S7E), which compromises the relationship between plant growth and soil microbial communities. However, K. obovata biomass was significantly correlated with RT in addition to environmental factors (i.e., soil water content and inundation frequency), which might be because K. obovata is generally regarded as a nutrient-limited species in subtropical coastal wetlands (34).

S. alterniflora invasion in coastal wetlands of China strongly influenced the soil microbial communities and their associated functions (38, 39). S. alterniflora can supply specific soil microorganisms with certain substrates, such as trimethylamine (63, 64). Here, we suggested that the changes of aboveground plant species were more closely associated with RT than with AT (Table S1B), due to RT’s narrower niche breadth and ecological preferences for certain habitats and resources (11, 65). With the continuous elevation of global sea level, global coastal wetlands are undergoing prolonged flooding (66), under which circumstance, S. alterniflora could continuously and aggressively expand into most coastal wetlands (67, 68). On the contrary, native mangroves, such as K. obovata, perform better at slightly higher elevations which are subjected to relatively less flooding. Such native mangrove habitats are fragmented and invaded by S. alterniflora due to increased frequency and duration of flooding as well as the intensification of human activities (69, 70). Subsequently, the dominancy of S. alterniflora could induce fundamental changes in the soil microbiome, which further influences the growth of plants (71). Moreover, it was reported that S. alterniflora invasion could suppress K. obovata biomass by up to 90% (29). Therefore, we suggest that protecting soil RT in coastal wetlands is crucial for maintaining and enhancing the functions of the ecosystem under the context of global environmental changes (e.g., biological invasion and deeper inundation). Future studies considering the community compositions and functions of root-associated microorganisms and plant endosymbionts could facilitate a deeper understanding of the responses of plant growth to global environmental changes.

In conclusion, this study demonstrates the mechanisms of AT and RT community assembly in response to environmental changes in coastal wetlands. S. alterniflora invasion in coastal wetlands increases the sensitivity of soil microbial communities to environmental changes. Furthermore, homogeneous selection is dominant in RT, while dispersal limitation primarily governs AT. In addition, the RT community is strongly associated with K. obovata biomass rather than S. alterniflora biomass. These findings provide a scientific foundation for better understanding the responses of coastal wetland ecosystems to global environmental changes from the viewpoint of microbial ecology.

## MATERIALS AND METHODS

### *In situ* mesocosm design.

An *in situ* mesocosm was designed and established in the Zhangjiang River Estuary (23°55′N, 117°26′E) in Fujian Province, China, according to previous studies (29, 72, 73). This area has a subtropical marine climate (annual mean air temperature at 21.5°C) with irregular semidiurnal tides (average range of 2 m to 3 m) (26). The mesocosm allowed us to investigate the soil microbiome along a fine-grained inundation gradient, eliminating the influences of other environmental changes and simulating the natural conditions.

In brief, three platforms, each consisting of two subplatforms (4 m in length by 2 m in height) that were placed in parallel along the direction from shoreward to seaward, were established in the intertidal creek in parallel at an approximately 3-m to 5-m distance from each other (Fig. 6A). A total of 11 elevations were obtained at a vertical interval of 25 cm (Fig. 6B), with the natural distribution of S. alterniflora and K. obovata completely covered by elevations 2 to 8 of the mesocosm. Elevation 1 was below the lowest elevation limit of the natural distribution, while elevations 9 to 11 were above the highest elevation limit. Two pressure transducers (HOBO water level, U20L-04) were installed at elevations 1 and 11 to monitor the inundation frequency. The inundation frequency was decreased with increasing elevation and ranged from 0.02% to 53.60% (Fig. 6C).

After the establishment of the mesocosm, six polypropylene buckets (25-cm inner diameter and 33-cm height) were placed on each elevation of each platform. Three holes with a diameter of 1 cm were poked at the bottom of each bucket and covered by a nylon screen (1-mm mesh) to facilitate the vertical exchange of seawater. Intact soil cores were collected from adjacent bare mudflats and placed into the buckets. All the initial soils had the same inundation frequency, salinity, and textures. Then, the six buckets on each elevation were evenly divided into two groups. In March 2017, three of the buckets were each planted with two K. obovata propagules; each of the other three were planted with one S. alterniflora seedling (Fig. 6D). Therefore, nine replicates per elevation of each plant were generated. The plants in this mesocosm were harvested in September 2018 (Fig. 6E), and the biomass of K. obovata and S. alterniflora was assessed using the method described by Zheng (74).

### Soil sample collection.

Soil samples were collected during the neap tide in September 2018. Although K. obovata and S. alterniflora in several buckets were dead or flushed away prior to the soil sampling, at least 5 replicates were randomly collected from each plant in each elevation. Three intact soil cores (with 0 cm to 15 cm in depth) surrounding the plants were collected from each bucket and then completely homogenized as one replicate (Fig. 6F). A total of 120 soil samples (58 for K. obovata and 62 for S. alterniflora) were collected in Ziploc bags, and the bags were immediately sealed with gummed tapes after removing fine roots and other debris (Fig. 6G). All of the soil samples were stored on ice during their transportation to laboratory.

### Measurements of soil physicochemical properties.

A total of 17 soil physicochemical properties were measured. Briefly, soil water content was determined gravimetrically (75). Soil NO_3_^−^ and NH_4_^+^ concentrations were measured after the samples were extracted with 2 mol/liter KCl by using a San^++^ continuous flow analyzer (Skalar, Breda, Netherlands). Soil salinity and pH were measured by using an FE20 digital meter (Mettler Toledo, Shanghai, China). Soil TC and total nitrogen (TN) were determined by using a 2400 II CHN elemental analyzer (PerkinElmer, Waltham, MA, USA), and then the carbon/nitrogen (C/N) ratio was calculated. Other soil elements (including P, K, Ca, Mg, Mn, Al, Fe, and Cu) were digested with 1:2:1 (vol/vol/vol) nitric acid (HNO_3_), hydrofluoric acid (HF), and perchloric acid (HClO_4_) on a hot plate and then determined by using an ICP Optima 8000 (PerkinElmer).

### Soil DNA extraction and high-throughput sequencing.

The total microbial DNA was extracted from 0.5 g fresh soil using the FastDNA Spin kit (MP Biomedicals, Santa Ana, CA), according to the manufacturer’s instructions. Bacterial 16S rRNA genes were amplified using the primers 515F (5′-GTGCCAGCMGCCGCGG-3′) and 907R (5′-CCGTCAATTCMTTTRAGTTT-3′) tagged with unique barcodes for each sample. High-throughput sequencing was performed on an Illumina MiSeq platform (Illumina, Inc., San Diego, CA).

### Bioinformatics analyses.

Raw read sequences were processed by the ASV method using the Quantitative Insight into Microbial Ecology 2 (QIIME2) pipeline (version 2019.10) (76). Sequences with poor quality (read length of <200 bp or average quality score of <25) were removed. Then, the filtered sequences were denoised by using DADA2 (version 2019.10.0) (77). Meanwhile, ASVs with fewer than 2 reads were discarded to avoid possible biases according to prior studies (17, 78). The SILVA database (https://www.arb-silva.de/, version 132) was employed for microbial taxonomy assignment. Finally, a total of 2,918,880 sequences were obtained from all of the 120 soil samples. Each sample was rarefied to 24,324 sequences (minimum) for downstream analyses. Then, all the ASVs were classified into 6 categories based on the cutoffs as described by recent studies (65, 79, 80): always abundant taxa (AAT), those with a relative abundance of ≥1% in all samples; conditionally abundant taxa (CAT), those with a relative abundance of ≥0.01% in all samples and ≥1% in some samples; always rare taxa (ART), those with a relative abundance of <0.01% in all samples; conditionally rare taxa (CRT), those with a relative abundance of <0.01% in some samples but not ≥1% in any sample; moderate taxa (MT), those with a relative abundance between 0.01% and 1% in all samples; and conditionally rare and abundant taxa (CRAT), those with a relative abundance ranging from rare (<0.01%) to abundant (≥1%). For the comparative study of AT and RT and to avoid confusions AAT, CAT, and CRAT were jointly counted as AT, and ART and CRT were jointly counted as RT (65, 81). Here, we mainly focused on the AT and RT, as MT were not detected in this study. The general description of AT and RT is shown in Table S1A in the supplemental material.

### Statistical analyses.

Two-way ANOVA was used to examine the effects of elevation, plant species, and their interactions on the alpha diversity of soil bacterial communities. Spearman’s rank correlation rho (ρ) was used to assess the relationship between soil physicochemical properties and bacterial alpha diversity. The significant difference between any two slopes was analyzed and compared by SMA regression analysis using the SMATR package in R software (82). A significant difference in bacterial alpha diversity between any two groups was examined by Wilcoxon rank sum test.

A PERMANOVA was conducted to determine the significant differences in soil bacterial communities among elevations and between plant species. In addition, a VPA was performed to assess the effects of plant species and soil physicochemical properties on bacterial community structure using the vegan package. Then, a Mantel test was used to determine the significance of the relationship between each soil physicochemical property and bacterial community structure. PCoA was performed based on the weighted UniFrac dissimilarities along environmental gradients. The spatial turnover rate (*Z* value) of soil bacterial communities was determined based on the distance-decay relationship between Sørensen similarity and environmental distance (83). The contribution of RT to the shifts in bacterial community dissimilarity was estimated according to Shade et al. (84). To assess the difference between AT and RT subpopulations, Spearman’s rank correlation ρ was used to assess the abundance-occupancy relationship of bacterial taxa between the log-transformed mean relative abundance of bacteria and the number of sites they occupied (85). The relationship between taxa’s niche width and their log-transformed mean relative abundance was also determined (86, 87). In addition, to better understand the biodiversity patterns and to explore their causes, compositional dissimilarities (beta diversity) among sites were subdivided into replacement and richness difference components (Podani family, Sørensen dissimilarities) using the adespatial package.

To further explain the patterns of beta diversity, Levin’s niche breadth indices of each bacterium were calculated by using the spaa package. The Orwin-Wardle resistance indices for the soil bacterial community were further calculated (88). In addition, the phylogenetic signals were estimated to examine if the environmental preference of a bacterial ASV was related to the phylogeny, reflecting the degree of phylogenetic conservatism in response to environmental gradients (21). To obtain the potential trait, we identified the ecological preferences of each ASV via the Spearman’s correlations between the relative abundances of ASVs and each soil physicochemical property (89). Next, Pagel’s lambda was applied to measure the phylogenetic signals for the environmental preferences of taxa using the phytools package (90, 91). The value of lambda is between 0 and 1, with larger values indicating a stronger phylogenetic signal.

A previously developed null modeling approach, based on the assumption of phylogenetically conserved environmental preferences of microbial lineages, was used to determine the relative influence of community assembly processes (2). This approach has been shown to provide robust estimates of the relative influences of different microbial community assembly processes in a range of ecosystems (1, 2, 20, 44). To observe phylogenetic conservatism among microbial ASVs, we tested phylogenetic signals in association with habitat using the “mantel.correlog” function with 999 randomizations (2, 42, 92). The community assembly processes were evaluated by calculating the NTI and betaNTI using the “ses.mntd” function in the picante package (93) and a previously developed null modeling approach (2), respectively. NTI values, the negative values of ses.mntd output, were used to evaluate the phylogenetic community assembly at a within-community scale, in which the positive and negative values indicated clustering and overdispersion of taxa across the overall phylogeny, respectively (50). According to Stegen et al., betaNTI is the number of standard deviations that the observed beta mean nearest taxon distance (betaMNTD) is from the mean of the null distribution (2, 42). A value of betaNTI of >2 or less than −2 indicates greater than or less than the expected phylogenetic turnover, respectively. The combination matrix of betaNTI values and Bray-Curtis based Raup-Crick (RC_bray_) was applied to estimate the relative contributions of homogeneous selection, variable selection, dispersal limitation, homogenizing dispersal, and undominated processes in governing community assembly (42). Pairwise betaNTI of less than −2 or >2 indicated homogeneous selection or variable selection, respectively. RC_bray_ of less than −0.95 or >0.95 indicated significant deviations from the null model expectation. |betaNTI| of <2 with RC_bray_ of less than −0.95 or >0.95 indicated a contribution of homogenizing dispersal or dispersal limitation, respectively. Otherwise (|betaNTI| < 2 and |RC_bray_| < 0.95), the shifts in community composition were undominated. The significance of the relationship between betaNTI and soil physicochemical properties was assessed by Mantel tests.

An SEM was used to evaluate the direct and indirect effects of inundation changes on K. obovata and S. alterniflora biomass. Prior to SEM analysis, classification random forest analyses were conducted by using the rfPermute package to identify the most important predictors that influenced plant biomass and bacterial community structures (represented by PCoA axis 1) of AT and RT, respectively. One thousand permutation replicates were then used to construct the null distribution and calculate the *P* values. All selected predictors were included in the SEM as independently observed variables. Since most of the variables were not normally distributed, the probability that a path coefficient differed from zero was assessed by using the bootstrapping method (94). Meanwhile, three metrics were used to test the goodness-of-fit of SEM (95): (i) chi-square test, in which a good fit is defined as 0 ≤ *x*^2^/df ≤ 2 and 0.05 < *P *≤* *1.00, (ii) Bollen-Stine bootstrap test, in which a good fit is defined as 0.10 < Bollen-Stine bootstrap and *P *≤* *1.00, and (iii) root mean square error of approximation (RMSEA), in which a good fit is defined as 0 ≤ RMSEA ≤ 0.05. Then, the standardized total effect of SEM attributes on plant biomass was calculated. SEM analyses were performed by using AMOS 21 (IBM SPSS Inc., Chicago, IL, USA). Statistical significance was determined by a *P* value of <0.05 for all analyses.

### Data availability.

All the obtained sequences were deposited in the NCBI Sequence Read Archive (SRA) with BioProject accession number PRJNA597962.
