# Supplementary figures and images for: Distinct Community Assembly Processes of Abundant and Rare Soil Bacteria in Coastal Wetlands along an Inundation Gradient
Source: mSystems. 2020 Dec 22;5(6):e01150-20. doi: 10.1128/mSystems.01150-20 (PMC7762797; doi:10.1128/mSystems.01150-20)

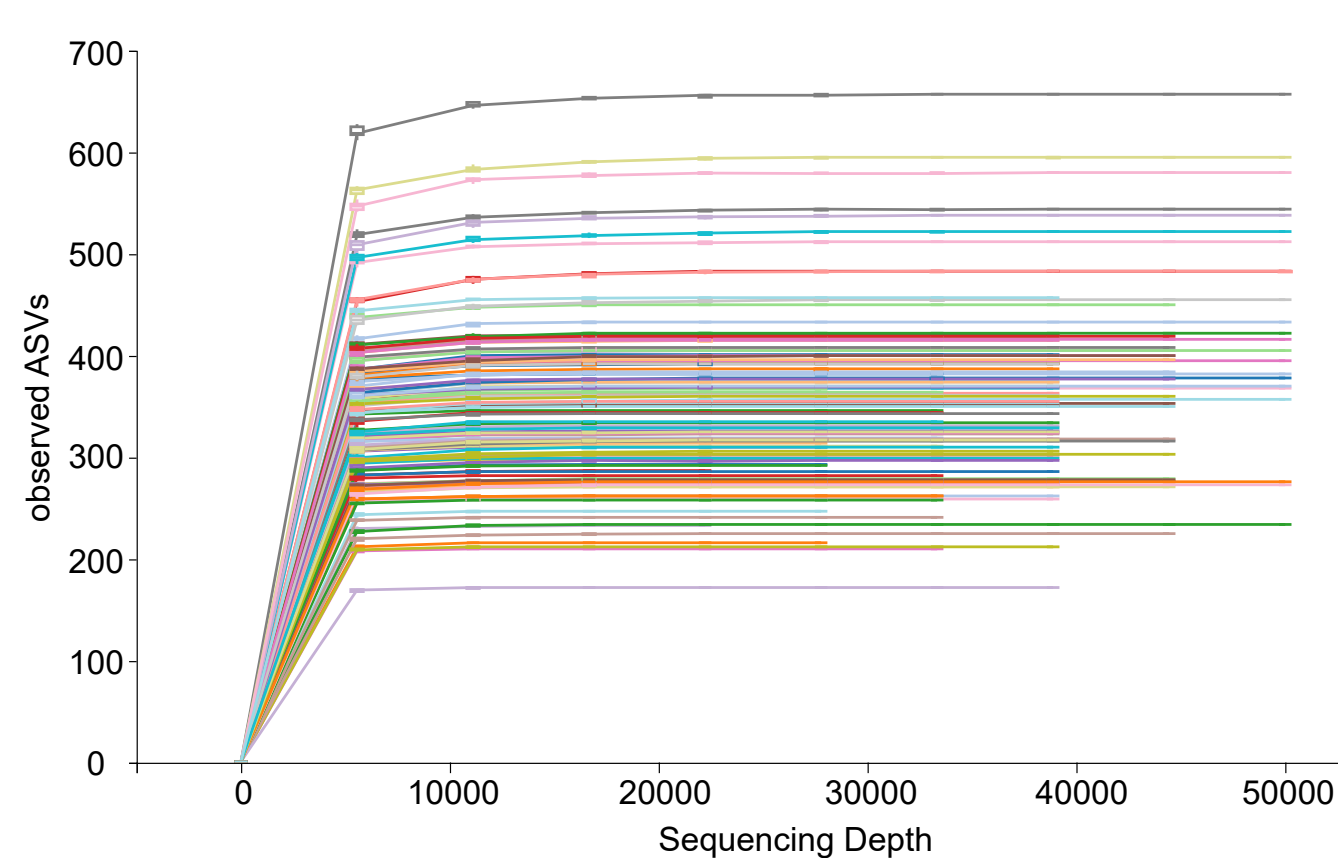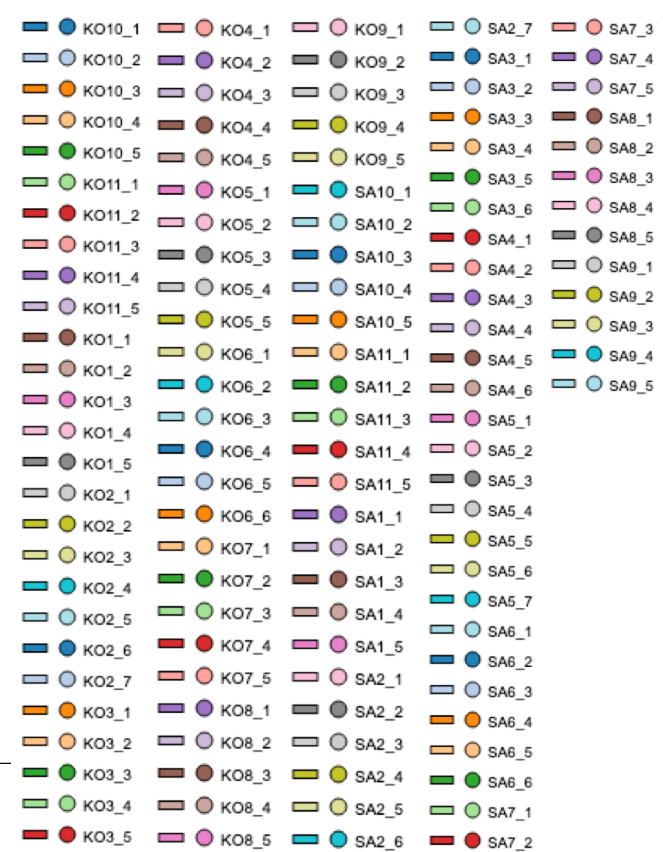

Supplement: FIG S1 [file mSystems.01150-20-sf001.pdf]

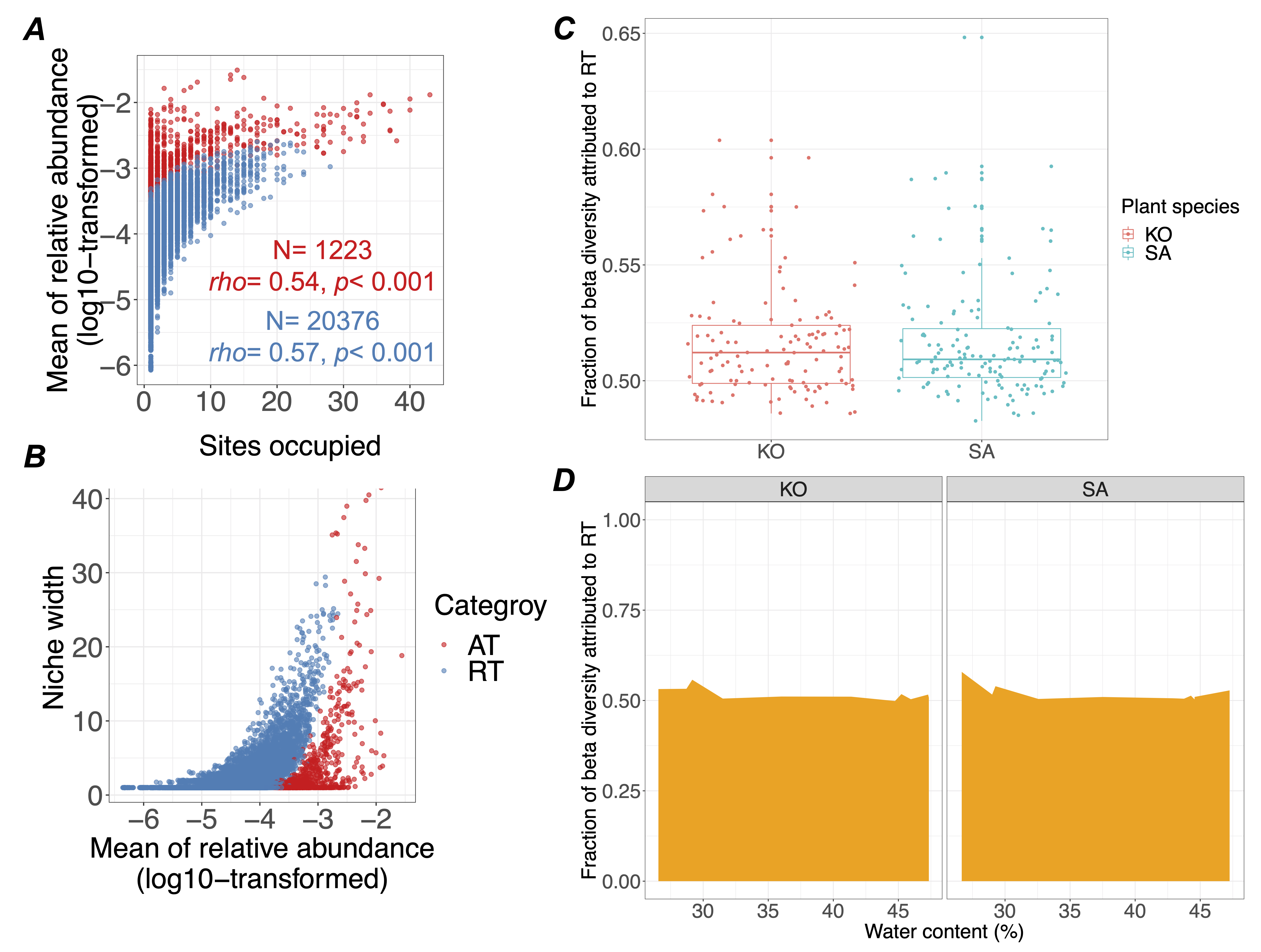

Supplement: FIG S2 [file mSystems.01150-20-sf002.tif]

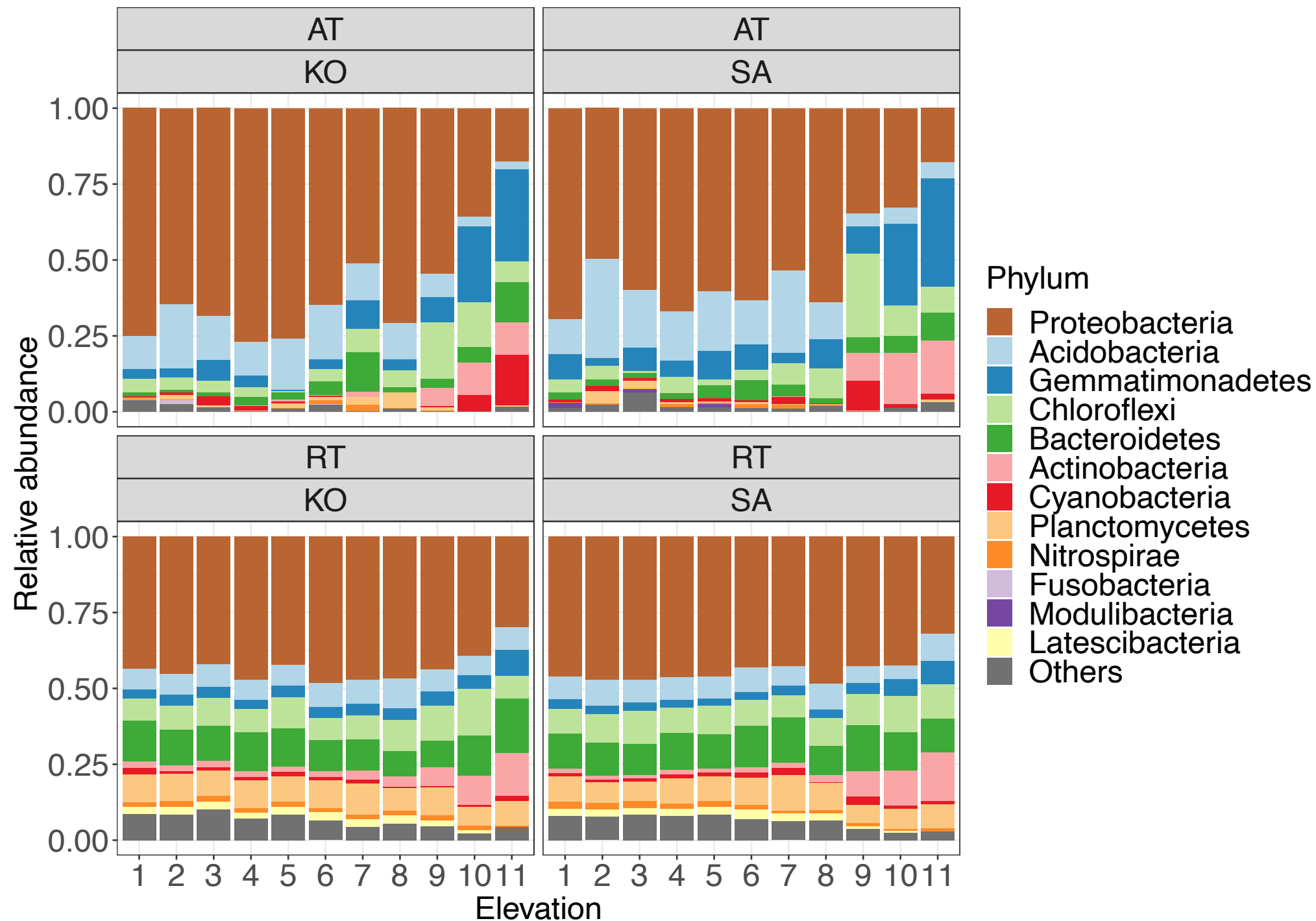

Supplement: FIG S3 [file mSystems.01150-20-sf003.pdf]

AT

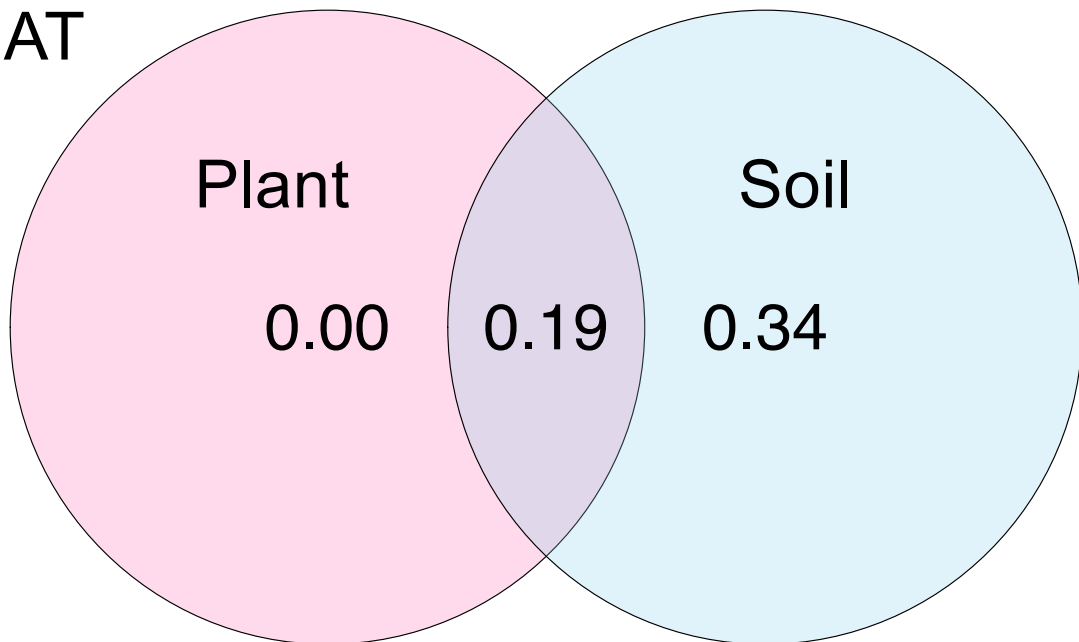

Residuals = 0.47

RT

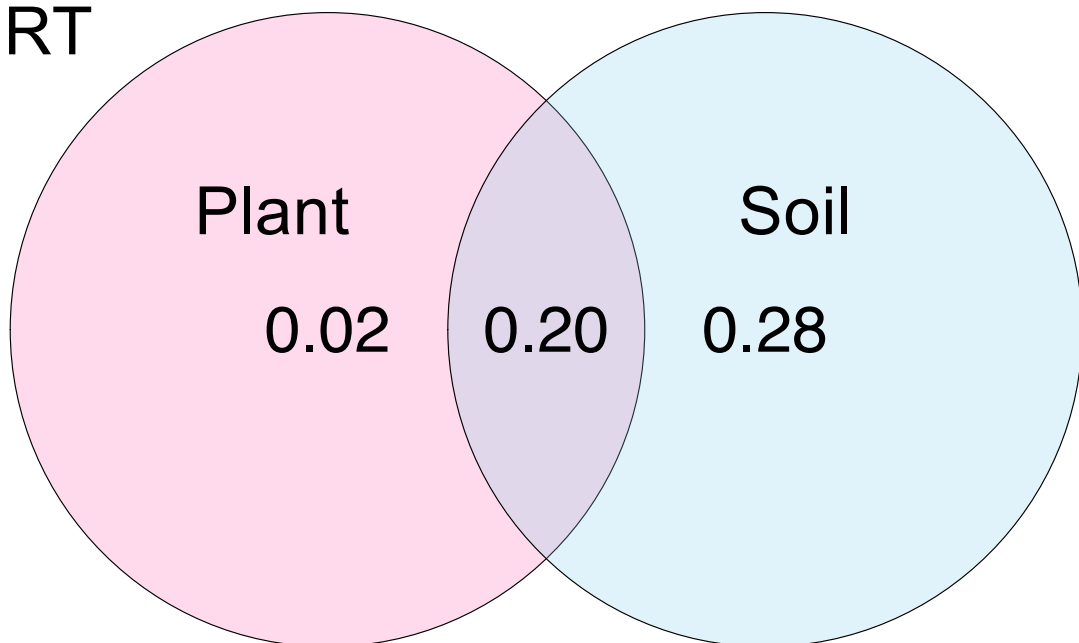

Residuals = 0.50

Supplement: FIG S4 [file mSystems.01150-20-sf004.pdf]

**A**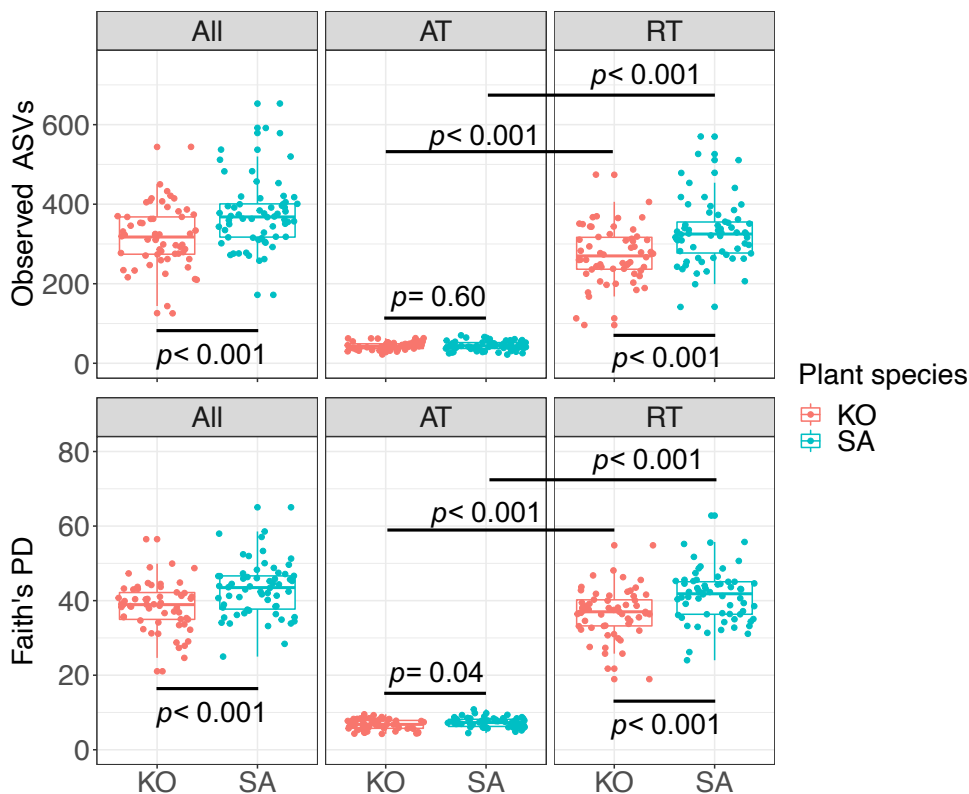**B**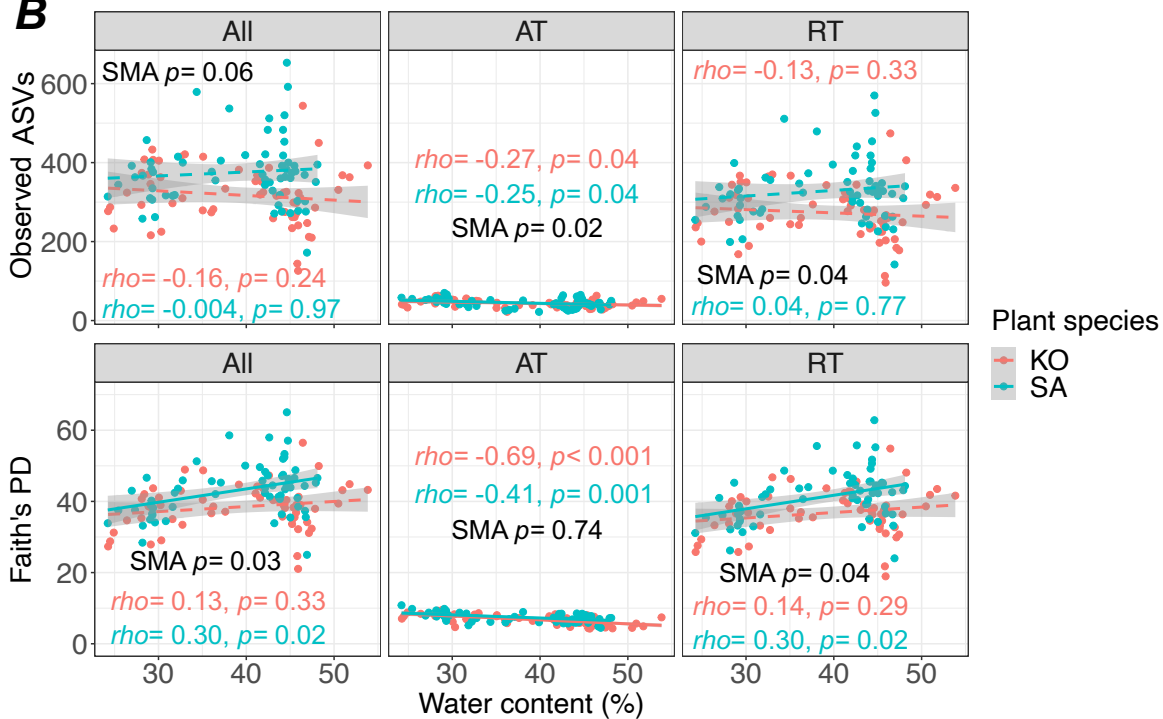

Supplement: FIG S5 [file mSystems.01150-20-sf005.pdf]

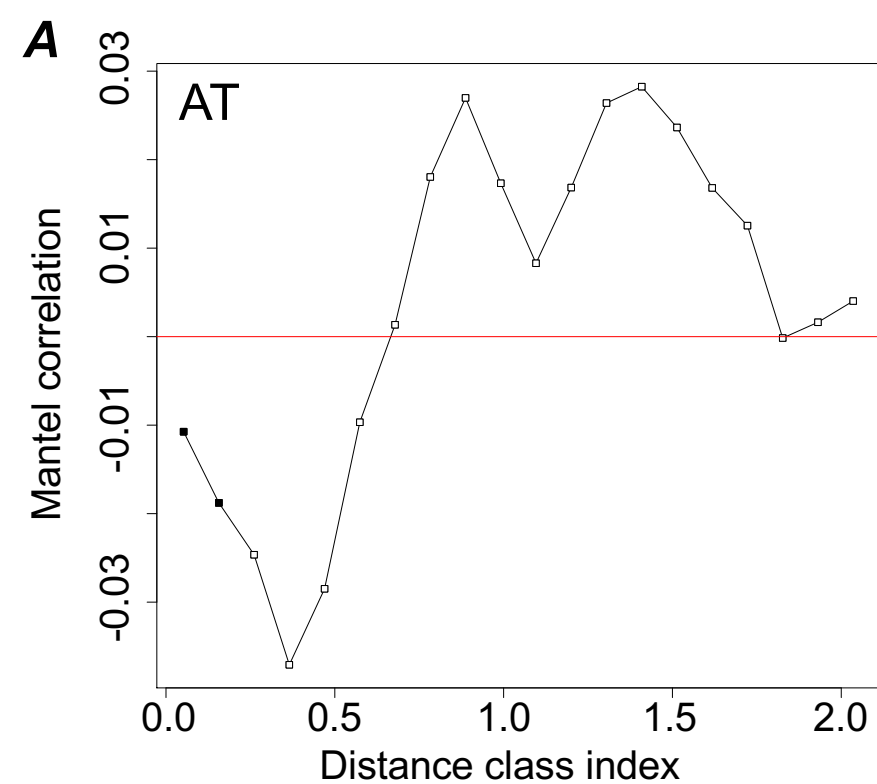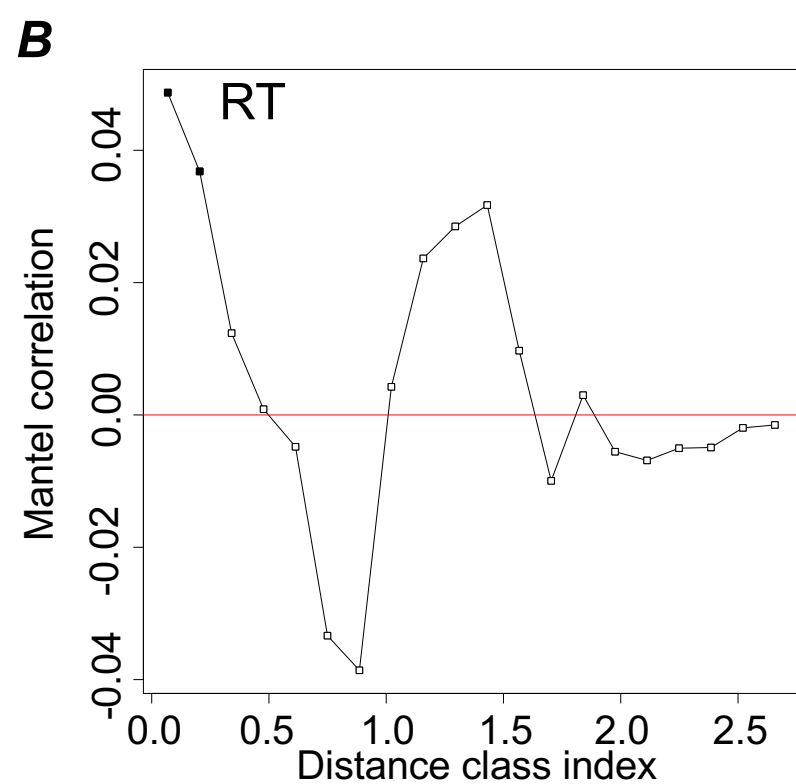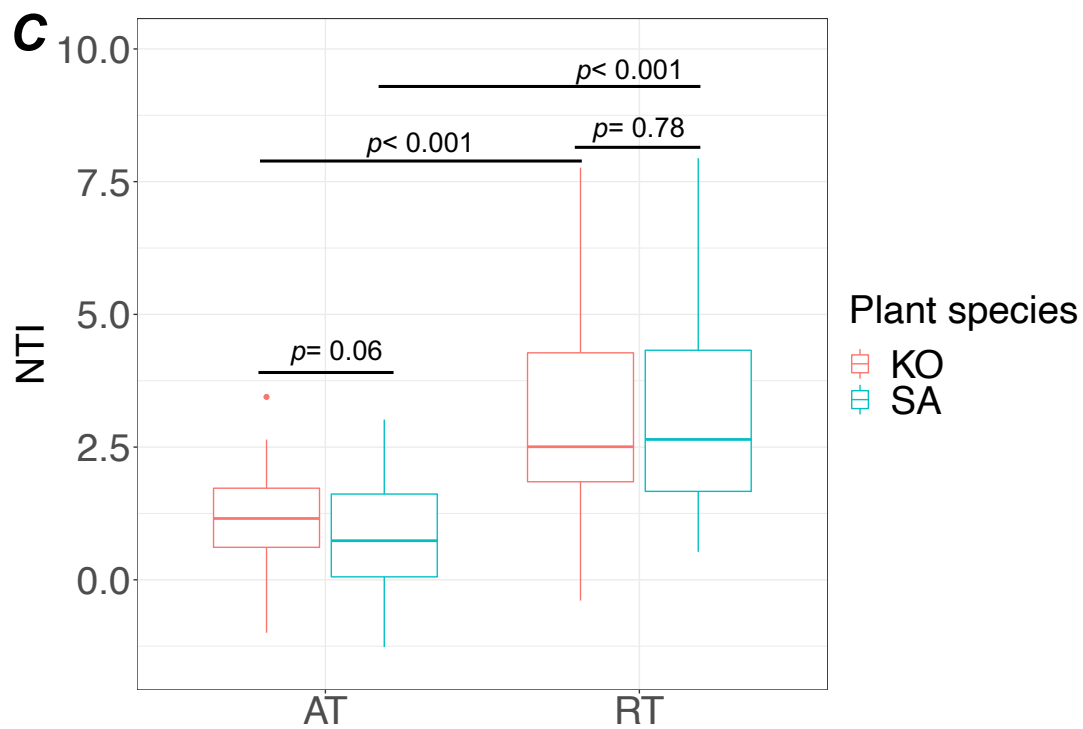

Supplement: FIG S6 [file mSystems.01150-20-sf006.pdf]

**A**

%IncMSE

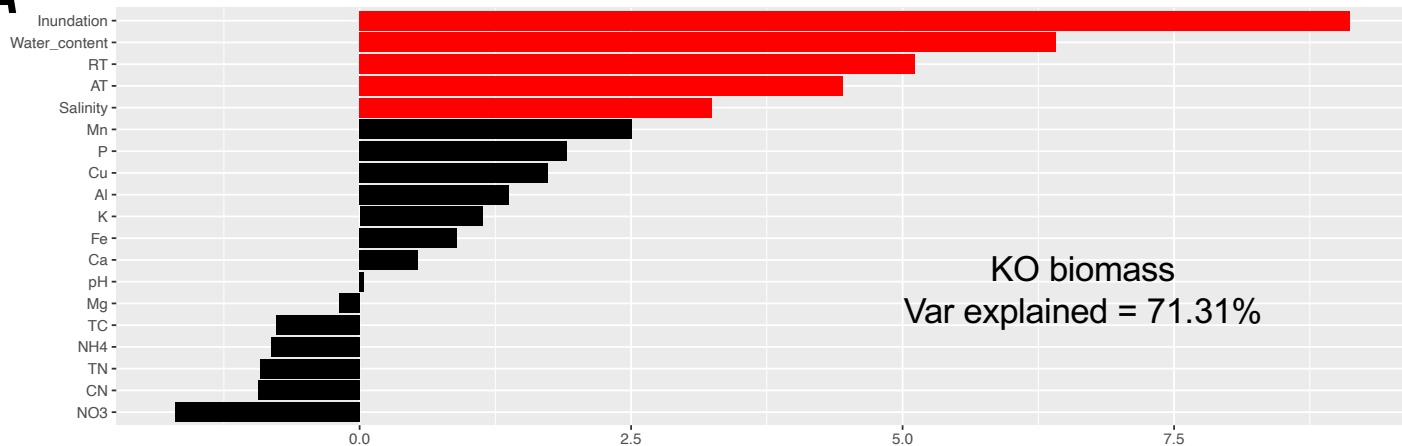**B**

%IncMSE

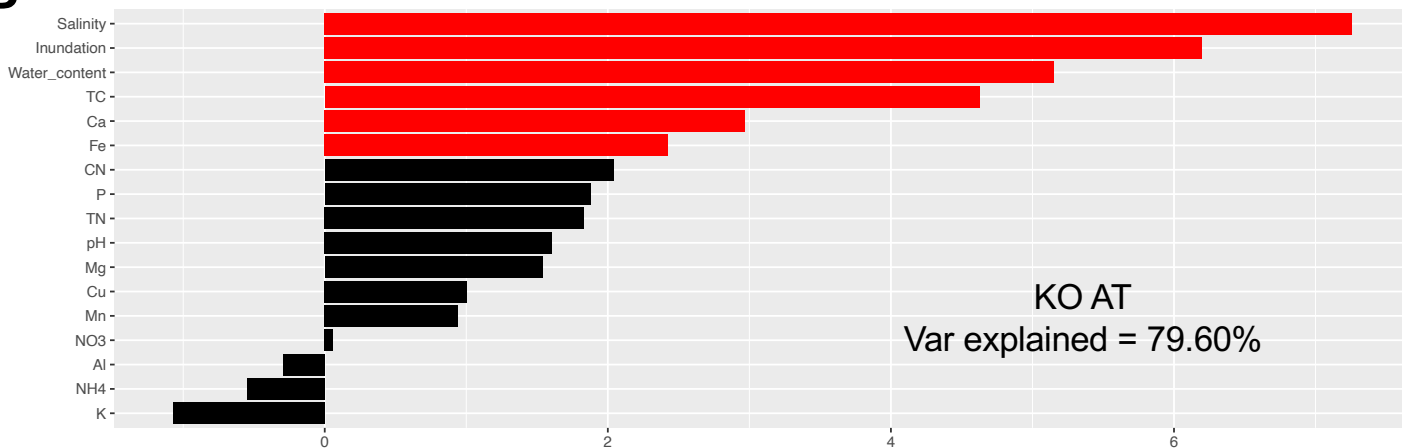**C**

%IncMSE

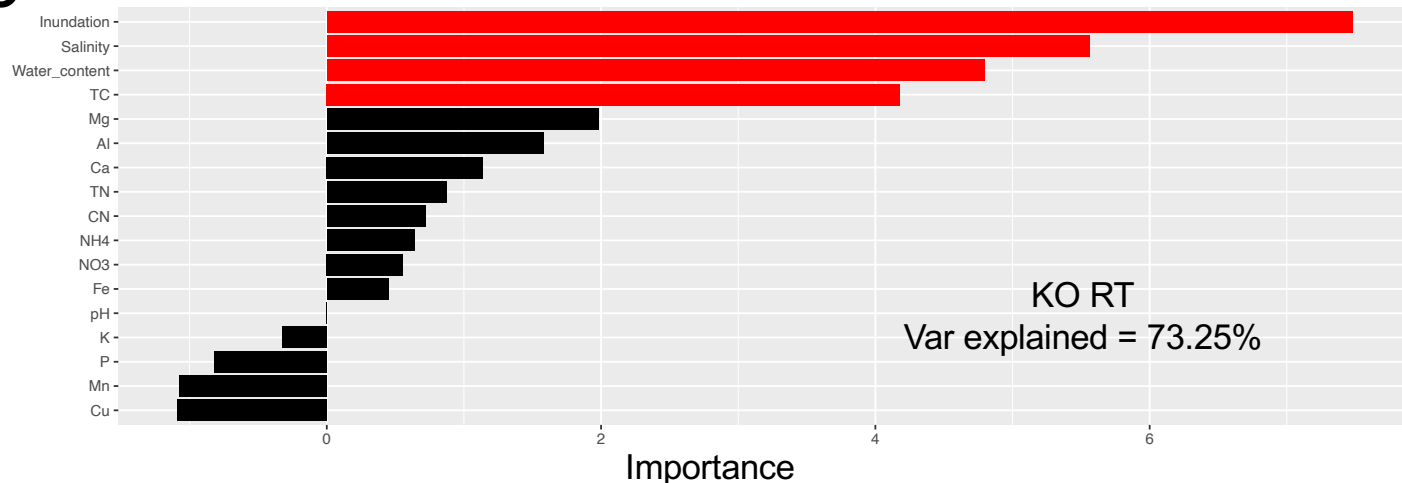

Supplement: FIG S7 [file mSystems.01150-20-sf007.pdf]

**A**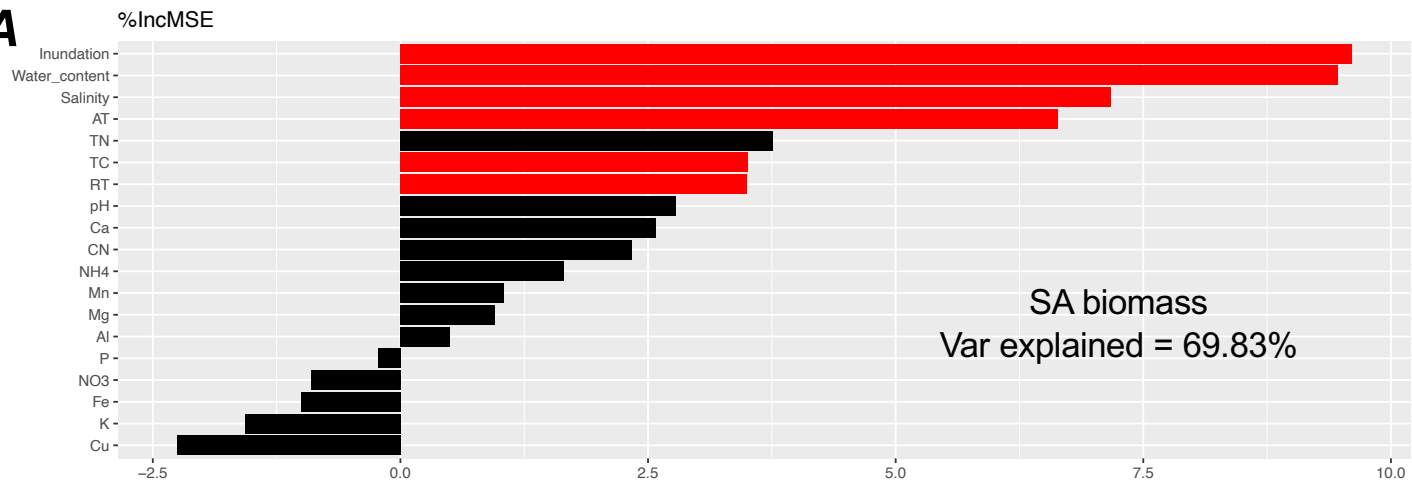**B**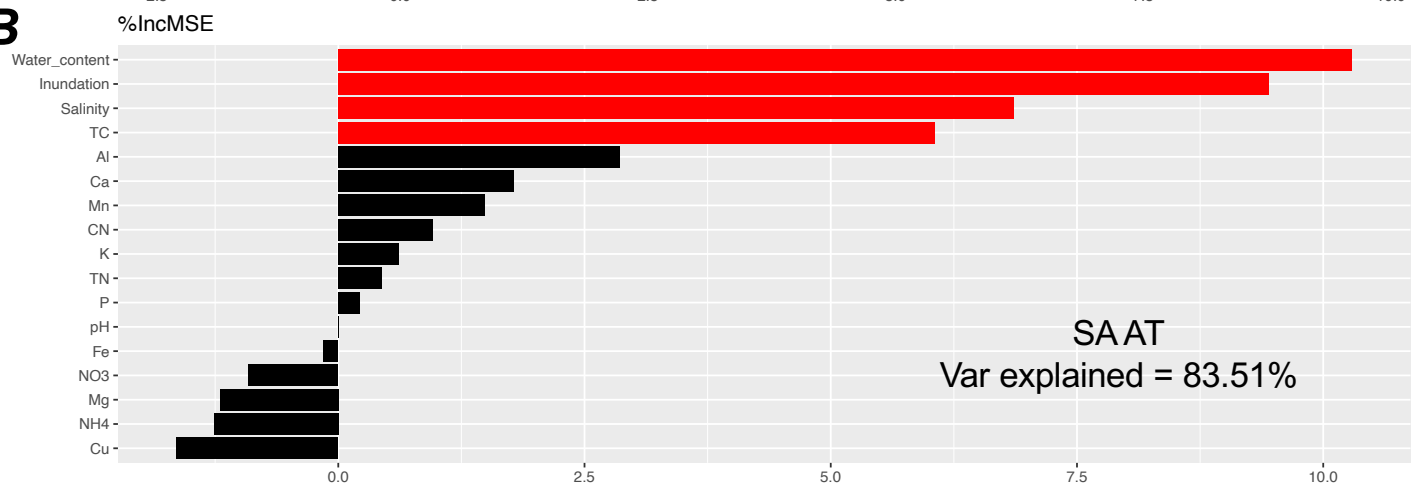**C**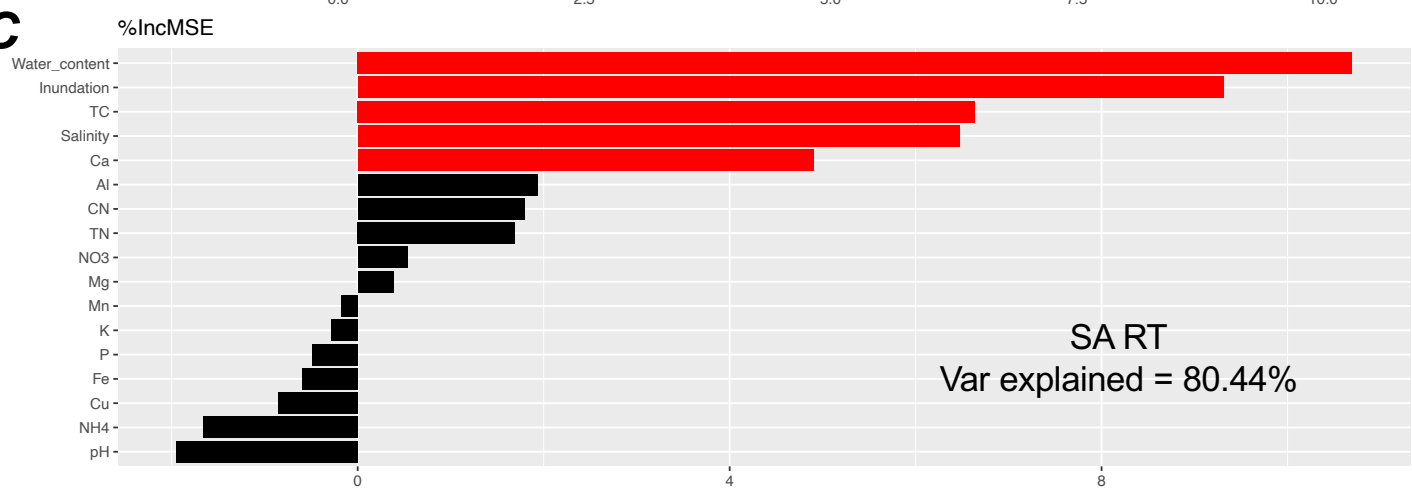

Importance

Supplement: FIG S8 [file mSystems.01150-20-sf008.pdf]

Standardized Total Effects

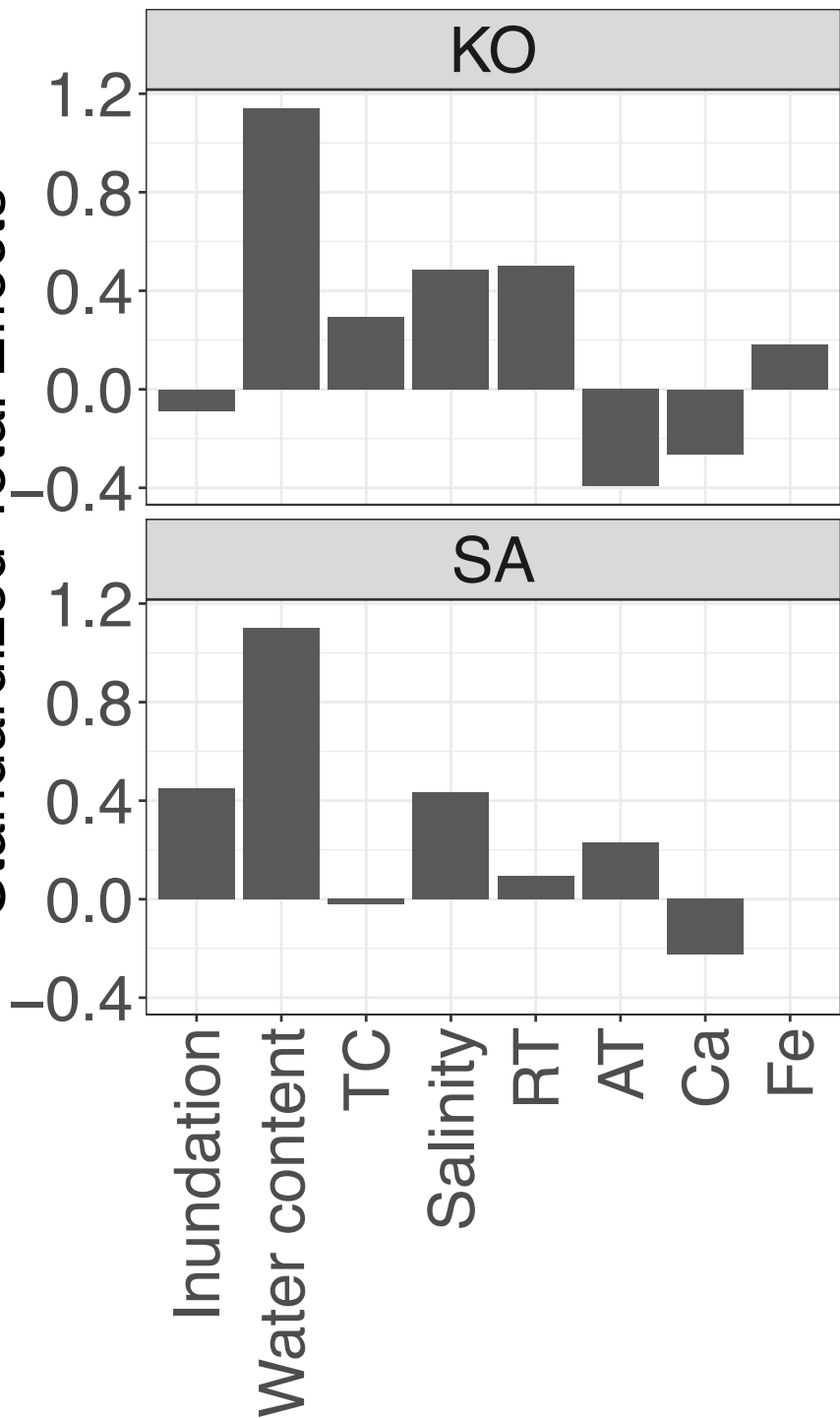

Supplement: FIG S9 [file mSystems.01150-20-sf009.pdf]
